# Performance Assessment of the Earthview BluBird Continuous Emission Monitoring System for Oil and Gas Facility Emissions Monitoring

**DOI:** 10.3390/s26185946

**Published:** 2026-09-20

**Authors:** James Maslanik, Kevin Yoon, Frederick Givhan

**Affiliations:** Earthview Corporation, 908 Dragon St., Dallas, TX 75207, USA; kevin.yoon@earthview.io (K.Y.); bear@earthview.io (F.G.)

**Keywords:** methane emissions, continuous monitoring system, point sensor network, oil and gas, controlled-release testing, METEC ADED, detection probability, emission quantification

## Abstract

The Earthview BluBird continuous methane emissions monitoring system participated in single-blind controlled-release testing in 2022 and 2024, with the goal of evaluating the leak detection and quantification capabilities of these metal-oxide point sensors under realistic field conditions. The METEC ADED test program consisted of natural-gas releases (“experiments”) at a simulated gas production facility, with each experiment comprising up to five simultaneous releases from different locations. Here, we analyze the results according to the detection of at least one release per experiment (“per experiment” detection) and the detection of all releases. Using METEC’s standard grading criteria, 91% of ADED 2024 experiments were detected; a substantial improvement compared to the 2022 results. Undetected releases during multi-release experiments, rather than undetected experiments, account for the large majority of missed releases. Detection correlated more strongly with release duration than with release rate. Detection rates and probability of detection improve when alternative classification criteria are applied. Sources were correctly identified at the equipment group level for 75% of single-release experiments. Actual and estimated methane-emission mass summed over daily to monthly intervals correlate significantly, with BluBird underestimating mass by 36% for single-release experiments. This underestimate is dominated by emission rate error rather than release detection capability.

## 1. Introduction

Methane emissions from oil and gas operations are estimated to have reached roughly 78 million metric tons in 2025 [1]. Using existing technologies, the oil and gas sector has the highest near-term potential for significant net reduction in methane release. This can be achieved by rapidly detecting and repairing emission sources at oil and gas production and distribution sites [2].

Among the many technologies being applied to this problem, Internet of Things-based continuous emission monitoring systems (CEMS) that combine inexpensive metal-oxide sensors with automated compensation for sensor drift and atmospheric effects are an attractive option [3]. These systems provide near real-time measurements, with the ability to detect and quantify dynamic, short-lived emission events [4,5]. This complements periodic monitoring technologies such as optical gas imaging and aircraft and satellite overflights by allowing for the rapid detection and repair of fugitive emissions that might otherwise persist for extended periods of time [6,7,8].

The strengths and limitations of commercially available, affordable, and practical CEMS are still being defined (e.g., [9,10,11,12]). General conclusions to date are that some relatively low-cost sensing systems are capable of reliably detecting methane emissions in the range of 5 to 10 kg/h at oil and gas sites [13]. The ability of CEMS to quantify methane emissions at levels suitable for some regulatory applications remains uncertain [11,14] and potentially limiting [15,16]. This is particularly true for the types of relatively low-cost sensors that would be practical and affordable for deployment at hundreds or thousands of operating oil and gas production sites [16,17]. The CEMS technology continues to improve, though, and it is important to weigh the performance of CEMS in terms of other candidate emission monitoring methods [9,18], including taking into account emissions that are missed due to intermittent leaks that would otherwise be detected by CEMS (e.g., [8]).

Testing programs are needed that can assess (1) the degree to which a CEMS is capable of detecting and reporting emissions at levels and time scales useful for reducing emissions; (2) whether the systems can provide at least a general idea of leak rates suitable for prioritizing leak detection and repair (LDAR) responses; and (3) how well such systems can operate consistently under a range of real-world field conditions. Comprehensive testing of emissions monitoring technologies is a difficult task, though, and optimal experiment design is still an area of research [10]. The “Advancing Development of Emissions Detection” (ADED; https://energy.colostate.edu/metec/aded/ [accessed on 17 June 2026]) project, conducted by the Colorado State University’s Methane Emissions Technology Evaluation Center (METEC; https://energy.colostate.edu/metec/ (accessed on 17 June 2026)) [9,18,19,20] helps to fill this need.

The primary goal of the ADED program, funded by the U.S. Department of Energy, is “to reliably test, quantify, and standardize the accuracy of next-generation emission detection solutions in highly realistic, controlled field environments”. During the three-month-long ADED test periods, controlled releases of natural gas are carried out at METEC’s simulated gas production pad at varying intervals and release rates, under all weather conditions and at different locations on the facility. Providers of emissions monitoring technologies pay a fee to METEC to participate in the ADED programs, and agree to a specific set of detection reporting rules. METEC then uses these “detection reports” to grade the performance of the technologies based on measures such as number of releases detected and missed, false detections, source-locating ability, and probability of detection as a function of gas release rates.

Reports and publications summarizing these ADED test results [18,19,20] are relied upon by regulators and oil and gas operators as definitive assessments of different emissions monitoring technologies. The weight given to ADED-based conclusions therefore means that providers and users of these technologies have a large stake in how ADED results are presented and interpreted, particularly since the technology vendors’ commitment of up-front fees, time and equipment to participate in ADED programs is considerable, which limits how often smaller companies can take part. Despite the relatively realistic design of the METEC facility and the comprehensiveness of the ADED testing plans, questions remain about how well results from published ADED testing (e.g., [18,19,20]) reflect actual performance at operating oil and gas sites [15]. Additional interpretation of ADED-provided technology performance data is therefore warranted.

In this study, we expand upon previous analyses [18,19,20] of the ADED experiments—focusing on the BluBird CEMS [21] (Earthview Corporation, Dallas, TX, USA). This CEMS combines inexpensive metal-oxide Internet of Things (IoT) sensors typically deployed as fence-line point sensors at oil and gas production facilities. Cloud-based machine learning and automatic baseline calibration allows for the detection of small changes in methane concentrations (see Section 2.1). These are converted to mass emission rates and used to determine if an emissions event is underway, and if so, the most likely source location on the facility. Once an event is identified, automatic alerts are sent to the facility operator. 

In the discussion below, we focus on four of the main aspects that define how well CEMS can contribute to reducing fugitive methane emissions from oil and gas operations: (1) emission detection, including time to detection and time to alerts; (2) emission event duration accuracy; (3) emission quantification; and (4) localization of the emission source. Here, we consider the performance of the BluBird CEMS during ADED single-blind field testing in February–May 2022 and 2024.

A specific research question as part of this analysis is the degree to which the ADED performance assessment is affected by experiment design. As described in Section 2.3, during the ADED testing periods, natural gas is released at different intervals, with up to five individual, simultaneous releases from one to several individual sources. Each of these release periods is referred to as an “experiment”. METEC’s standard grading and assessment methods prior to 2025 (e.g., [9,18,19,20] and https://metec.colostate.edu/aded-testing-results/ accessed on 18 June 2026) do not differentiate between whether a monitoring technology entirely misses a period when one (or more) leaks were present versus whether the technology detected that at least one release was underway during a multiple-release experiment but failed to identify all of them. Essentially, the issue at hand is how best to use the ADED results to understand and differentiate between how well an emissions monitoring technology detects multiple, simultaneous leaks on a site as well as the ability to detect that at least one leak is present on a site. Here, we refer to the latter as detection of an “active site”. The term “site-level” detection is sometimes used in this context, but this implies that a technology may not be able to say more than that “a leak is present” when in fact CEMS like BluBird usually also provide additional details about leak location and the possibility of multiple leaks.

To understand the potential significance of this distinction when judging how well a technology meets the key objectives of detecting and reporting leaks, let us consider the following case of two sensor technologies, Technology A and technology B, operating during two consecutive ADED experiments. The first experiment consists of four simultaneous releases and the second experiment consists of a single release. Technology A correctly determines that at least one leak is present during both experiments but fails to uniquely identify two out of the four releases present during the first experiment. In the real world, if the experiments represented two different oil and gas production pads, this would translate to Technology A alerting the operator that their sites both have leaks, but it underestimates the likely number of concurrent leaks at the first site. In contrast, Technology B correctly identifies all four releases in the first experiment but fails to detect a leak during the second experiment. In this case, the operator would not have been alerted to a leak at the second site.

Under METEC’s standard grading of ADED results based on the original ADED test protocol used prior to 2025, Technology A would be assigned two “false negatives” (FN) and three “true positives” (TP). Technology B would be assigned just one FN, along with four TPs. If no further explanation is provided to readers of ADED results papers, they would likely conclude from the above report that Technology B is the better technology since its score is four TPs and one FN compared to Technology A’s three TPs and two FNs. What is not apparent from this simple count of TPs and FNs is that Technology B missed one active-site leak entirely, whereas Technology A detected active-site leaks in both experiments. In real-world terms, for this scenario, Technology A would result in a greater overall reduction in emissions since an LDAR team dispatched to both sites would likely find the two additional releases that Technology A did not report, whereas failing to detect an active-site leak at all, as in the case of Technology B, would allow the leak to continue unabated. 

The takeaway from this is that there is a need to know how effective a technology is at detecting whether a leak is present on a site, in addition to how well it detects multiple leaks. Here, we address this with respect to the BluBird CEMS by analyzing results on a “per experiment” detection basis in addition to the “all-release” basis used in METEC reports to date (see Section 2.4.3). In addition, to investigate how multiple simultaneous releases might affect detection and quantification, we adopt the approach used by [22,23], in which experiments that consist of only one release are examined as a separate set.

A secondary but still significant issue in terms of detection technology assessment pertains to METEC’s ADED report-classification rules. To be assigned as a true-positive detection, for ADED programs prior to 2025 METEC required that the participant’s reported start time for a detection be no more than 20.0 min earlier than METEC’s recorded start time. As will be seen, this interacted with the BluBird 2.0’s 10-min time-marching approach used for automated leak detection and reporting, which introduced a slight early start-time offset in the detection reports that resulted in the rejection of reports that otherwise correspond well with METEC releases. In the Results section (Section 3), we focus on BluBird performance as defined using the standard METEC classification criteria, and treat this as the primary performance benchmark. However, we also augment this with an analysis of the sensitivity of results to alternative classification rules that allow for a slightly earlier detection-report start time and that consider the temporal overlap of detection reports with release events. Since additional detections that result from the use of these alternative classifications are due to this modified grading criteria rather than to improvements in system performance, we refer to these cases as “potential” detections.

METEC has since revised its ADED testing protocol to address, among other aspects, the two situations discussed above: the issue of any-release versus all-release detection and the effects of revised or relaxed detection classification criteria [24]. However, as far as we are aware, METEC has not published analyses of earlier ADED test results that apply these new criteria to earlier ADED results.

This paper is organized as follows: Section 2.1 introduces the main elements of the BluBird system that are relevant for the presented analyses, including the detection and reporting methodology used for participation in the ADED 2022 and 2024 programs. Section 2.2 reviews the METEC testing configuration and assessment protocols. Section 2.3 describes the ADED programs and test-result data sets, emissions release strategies, and performance grading criteria. Analytical methods are summarized in Section 2.4, including the introduction of alternative performance classification metrics intended to comprehensively represent release detection performance.

Test results are presented in Section 3, focusing mainly on ADED 2024 but referencing the ADED 2022 results where relevant. We begin with the fundamental question of how well the BluBird CEMS detected and reported start and end times of individual ADED natural gas releases (Section 3.1). Section 3.1 also makes the case for including the additional alternative classification rules to assign detection reports as true-positive detections versus missed releases (false negatives) or false positive detections.

Rates of detection as functions of gas release rate and release duration are given in Section 3.2. Section 3.3 describes statistically derived probability of detection (PoD), considers the effects of point sensor network (PSN) density, and introduces the concept of a time-based PoD. Minimum detection level, detection of multiple releases and time to detection are treated in Section 3.4, Section 3.5 and Section 3.6. Emission rate quantification and total methane mass emitted over time are investigated in Section 3.7. Section 3.8 addresses the determination of emission source locations. Factors influencing release detection and quantification, including the potential effects of residual, lingering gas on the METEC site, are considered in Section 3.9. The sensitivity of results to classification criteria appears in Section 3.10.

Among the key findings of this study are: (1) the BluBird system demonstrates, across the full range of release rates tested and based on METEC’s standard grading criteria, a strong ability to detect when at least one leak (i.e., an ADED release) is present on the METEC site at leak rates well below 5 kg/h; (2) failure to detect all releases during multi-release experiments, rather than failure to detect that at least one release was underway, accounts for the large majority of missed releases; (3) the timing and duration of releases are accurately determined; (4) for the range of release rates and environmental conditions seen during ADED 2024, BluBird’s detection of releases correlated most strongly with release duration and wind conditions, with weaker correlation with release rate; (5) the 90% probability of detection (PoD) estimate for detecting the presence of a leak (i.e., 90% PoD for detecting an actively-leaking site) ranges from 0.64 kg/h for the detection of experiments, compared to greater than 10 kg/h for the detection of all releases (the standard measure used by METEC); (6) estimates of total emitted methane mass correlate significantly with actual emission totals, but with a negative bias; and (7) time averaging of release rates over daily to monthly periods improves the temporal match with actual emissions. The underestimation of total emissions is due mainly to emission rate error rather than to undetected releases, which suggests that future work should prioritize improving rate calculation accuracy rather than detection capability. A main takeaway from this study is that analyses of test results for emissions monitoring technologies should consider, and report on, the ability to detect active-site releases in addition to the ability to detect multiple, simultaneous releases. Finally, the utility of the PoD measure is considered, along with alternative ways of viewing PoD when comparing continuous monitoring systems with periodic monitoring technologies.

## 2. Materials and Methods

### 2.1. BluBird System Overview

**Figure 1:**
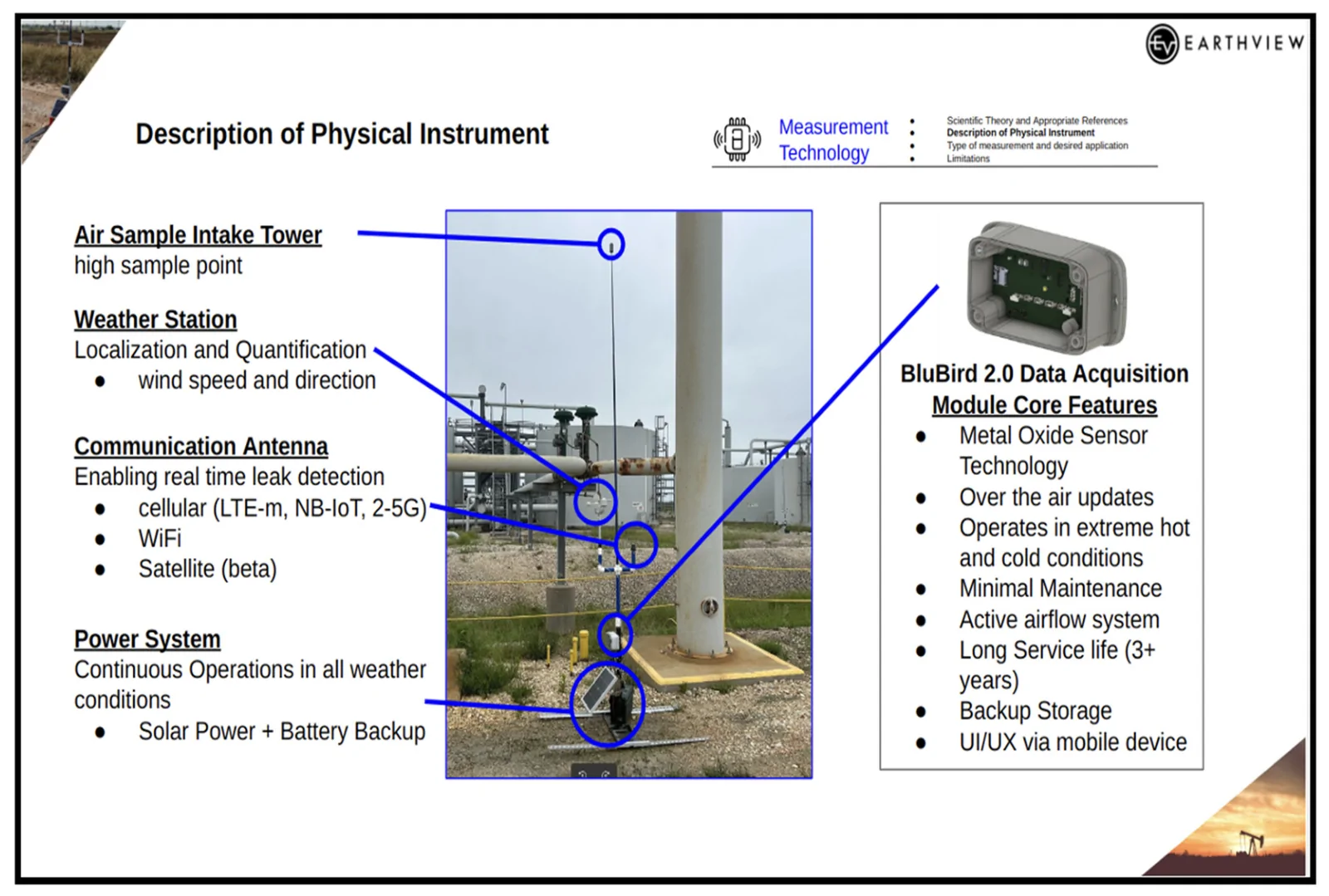
The Earthview BluBird 2.0 sensor node, showing stand, solar panel and backup battery, instrument enclosure, communications antenna, wind anemometer, and adjustable telescoping air-intake mast. At present, over 450 BluBird sensors are deployed at more than 100 operating oil and gas production facilities across the U.S.

The Earthview BluBird gas monitoring system (U.S. patents US 12,163,939 B2; US 12,216,103 B2; Earthview Corporation, Dallas, TX, USA) [21] includes three main subsystems: (1) a sensor network consisting of one to multiple solar-powered autonomous IoT field sensor nodes that operate as point sensors (Figure 1) deployed at a site (typically in a “fence-line” configuration); (2) a suite of cloud-based data acquisition and analysis software that converts sensor readings to methane concentrations and then to emission rates using the node-reported local wind information; and (3) a data access and display dashboard for site management, graphical analysis, time series presentation, and emission summaries. The number of nodes used on a site is dictated by the site complexity, wind patterns, and expected potential for multiple simultaneous emissions from different locations. The choices regarding how many nodes to deploy and where they are to be placed on a site are driven by the fact that point-sensor CEMS are unable to measure 100% of emissions all the time [25]. The node configuration therefore depends on clients’ priorities and budget, site characteristics, and regulatory requirements.

BluBirds use an array of three metal-oxide semiconductor (MOS) gas sensors, regulated air flow via a pump, an adjustable-height mast with air intake, solar power and backup battery (Figure 1). The specific MOS sensors used in the BluBird include the Figaro Taguchi Gas Sensor (TGS)2600, Figaro TGS2611-E00, and Figaro TGS2602 (Figaro Corporation, Mino, Osaka, Japan). TGS2600 responds to methane and non-methane VOCs. TGS2602 is affected primarily by non-methane VOCs, while TGS2611-E00 has a similar response to methane to TGS2600, but includes a filter to reduce the influence of non-methane VOCs. Fusing these data together enables some compensation for gas mixtures common at certain oil and gas facilities. Both TGS2611 and TGS2600 and have been shown to be capable of measuring methane concentrations at the low ppm level [26,27,28,29], albeit with calibration and adjustments for environmental effects, as described below.

The basic operating principal of this and other point-sensor CEMS is that natural gas that is emitting from one or more sources on the site is transported by wind or diffusion from the source to one or more BluBird sensors. Wind speed, wind direction, humidity and air temperature are measured at each sensor in the PSN and transmitted to the Earthview cloud site at approximately 30-s time intervals. The full process of going from raw sensor measurements to methane concentrations consists of the following main steps: (1) receive sensor data and perform quality checks; (2) predict “clean-air” MOS resistance (R_o_); and (3) calculate methane concentration from the ratio of observed resistance (R_s_) to R_o_ using a response function that captures the relationship between resistances and methane concentrations. The conversion from concentrations to mass emission rate is achieved using an inverse form of dispersion modeling, as summarized below.

When a target gas such as methane interacts with an MOS sensor, it results in a change in the sensor’s electrical resistance, which can be measured. The devices are typically highly sensitive and have a rapid response time of less than one second. However, they are engineered to respond to particular classes of gas rather than a single species and are sensitive to changes in atmospheric water content [28,29]. The BluBird data processing must therefore account for these factors as well as others such as sensor drift or degradation over time in order to detect methane concentrations of a few ppm or less in open air. Enhanced operating methods such as temperature cycling of the MOS sensors combined with machine learning [30] can improve the gas selectivity of standard sensors but require more advanced circuitry (and cost) than is presently implemented in the current BluBird system. 

Our approach for addressing these influencing factors is to generate time-evolving statistical models (digital twins [31]) of each individual sensor, specific to each BluBird unit in the field. A set of processing steps is first used to identify observations at each sensor unit that are likely to represent atmospheric background conditions (i.e., “clean-air” cases). The models are fit to these observations, yielding an estimate of the expected R_o_ at each time step that best fits the measured air temperature and humidity, as well as other, indirect characteristics of the atmospheric background and the sensor itself that are captured by the digital twin models. Stated another way, this process establishes a baseline resistance that the sensor should exhibit in the given conditions, minus the influence of methane. The resulting ratio between R_s_ and R_o_ is taken to represent the portion of the resistance change that is due to the presence of methane.

This ratio is then used to calculate the methane concentration based on response curves relating R_s_/R_o_ to concentration (e.g., [32,33,34,35]). A set of response curves has been developed in-house for different compositions of pure methane and natural gas, using a variety of data sets, including results in the published literature, lab experiments with calibration gases, co-located field measurements with air monitoring instruments and a research-grade methane reference-grade sensor. Overall, we find that these response functions are quite consistent between different BluBird units and are sufficiently stable over time.

**Figure 2:**
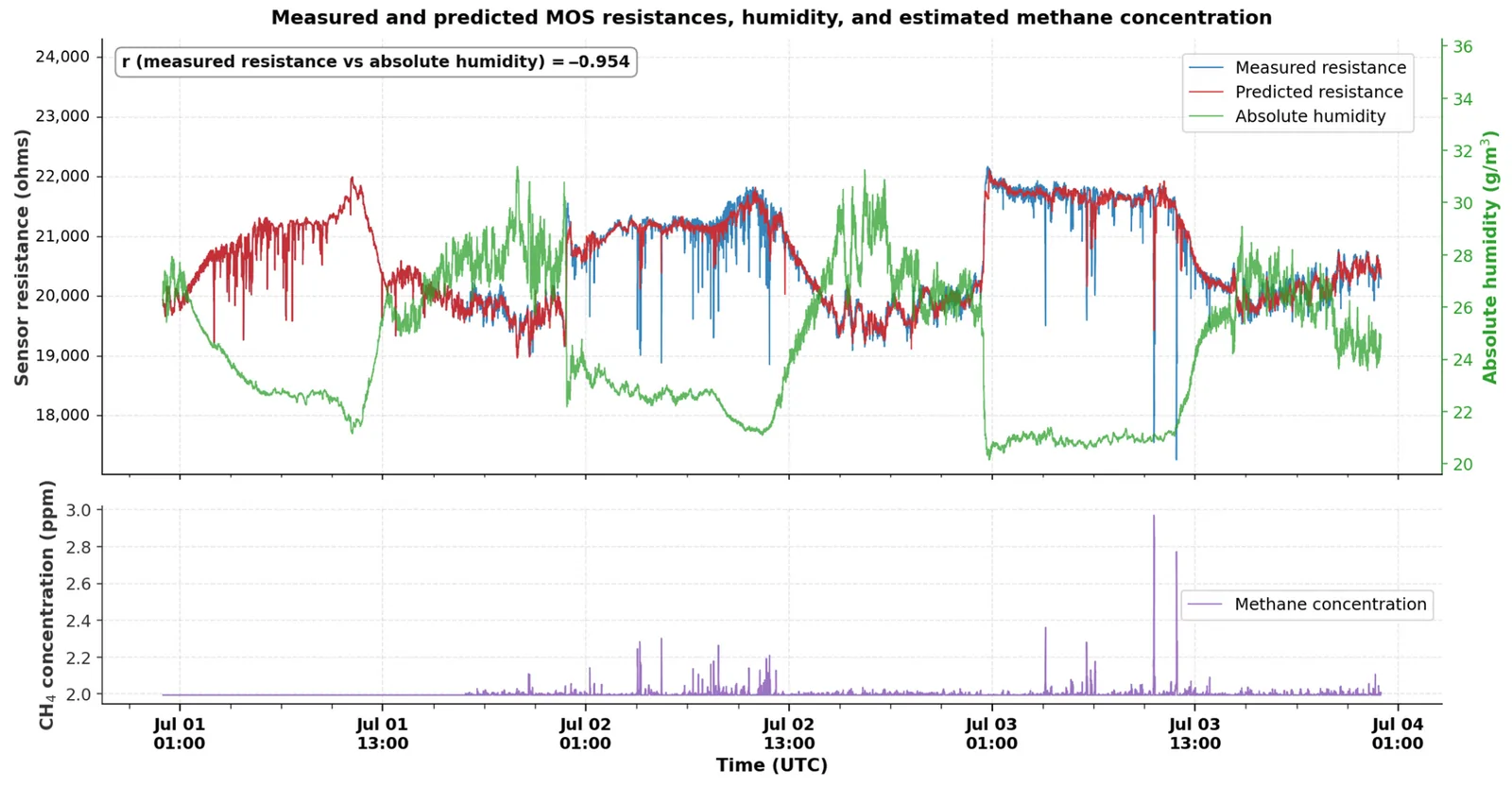
Observed and model-predicted MOS (TGS2600-E00) resistances and atmospheric humidity (top panel) and methane concentrations derived from the ratio of observed to predicted resistance, where the predicted resistance is the resistance expected for the time step if no additional methane was present (e.g., background atmospheric conditions). These data were collected at an operating oil and gas pad over a three-day period.

Figure 2 illustrates the role of humidity in the MOS measurements, along with the results achieved by applying the data adjustments introduced above. The figure includes a time series of observed MOS R_s_ (Figaro TGS2611-e00 data in this case), R_o_ (digital twin model-predicted resistance), and humidity in the top panel, along with the methane concentrations estimated from the resulting R_s_/R_o_. Of particular note is the typically strong correspondence between humidity and MOS resistance (a correlation coefficient of −0.93 in this case), and the intermittent drops (negative spikes) in resistance. Such spikes are typical of the presence of wind-transported methane. In the example shown here, if the atmospheric effects are not accounted for (as has been done to yield R_o_), the time series would yield apparent and false variations of several ppm of methane due to the humidity variations alone. In some situations where large and rapid changes in humidity occur, this error associated with humidity could easily trigger false leak alarms. While the likely effects of methane in this example appear as distinct spikes that could be easily extracted from the time series using signal processing, this “spikiness” depends on variability in wind and emissions conditions and cannot always be relied upon on its own.

As noted earlier, a CEMS typically requires that emissions be transported from a source to the sensor via wind transport and/or lateral diffusion. To convert from measured concentrations to estimated emission rate at the source, we apply an inverse version of the standard Gaussian plume model to calculate methane leak rates for most situations. This is a widely used approach (e.g., [36,37,38]). An overview of its advantages and disadvantages for real-time screening applications is provided by [39]. The applicable theory is that knowledge of local atmospheric conditions (winds, surface-layer stability, turbulent mixing potential, etc.) along with knowledge of the relative positions of the sensor and emission source, can be used to predict the degree of dispersion of methane along its travel path from source to sensor. Our modeling implementation follows the methodology outlined in EPA’s Other Test Method (OTM) 33A applied to oil and gas pad sampling [36,37,38,40,41].

The standard inverse form of the Gaussian plume model is as follows:

$$q=\frac{2\pi u\sigma_{y}\sigma_{z}\;c\left(x,y,z\right)}{exp\left(-\frac{y^{2}}{2\sigma_{y}^{2}}\right)\left[exp\left(-\frac{{\left(z-H\right)}^{2}}{2\sigma_{z}^{2}}\right)+exp\left(-\frac{{\left(z+H\right)}^{2}}{2\sigma_{z}^{2}}\right)\right]}$$

$$\sigma_{y}=A_{y}x+B_{y}\;\;;\;\;\sigma_{z}=A_{z}x+B_{z}$$

*q*: emission rate [g/s]

*c*: methane concentration [g/m^3^]

*u*: wind speed [m/s]

*H*: vertical distance between sensor and leak source [m]

*x*: horizontal distance between sensor and leak source [m]

*y*: horizontal distance from plume center [m]

*z*: vertical distance from plume center [m]

$\sigma_{y}$: *y* direction dispersion coefficient

$\sigma_{z}$: *z* direction dispersion coefficient

*A_y_*, *B_y_*, *A_z_*, *B_z_*: dispersion coefficient equation parameters for linear fits to OTM-33A dispersion tables (see Table 1).

In the OTM-33A appendices, $\sigma_{y}$ and $\sigma_{z}$ are linear functions of plume travel distance and stability. We fit equations to the set of coefficients for each of the seven stability classes to provide *A_y_*, *B_y_*, *A_z_*, and *B_z_*. (Table 1). Stability classes are estimated using the Pasquill method (e.g., [42]), based on site-measured wind speed and estimated solar insolation, with three levels of solar intensity defined (<300 W/m^2^, 300–700 W/m^2^, and >700 W/m^2^), which relate to local surface-layer stability.

The model assumes that transport of a gas in the x direction is dominated by wind advection [43]. At low wind speeds (typically taken as <2 m/s) or in calm conditions, this assumption begins to break down, and diffusion in the x direction becomes dominant [44,45]. In these situations, we instead use a modified version of the Gaussian model [46].

The standard-form Gaussian plume model formulation (and more specifically, the dispersion rates used in the model) is not well tested for short distances of several meters or less. In situations where the distance between the sensor and the leak source is small (less than 9 m), we instead use a simple cube model, which [47] argues yields reasonable results for contaminant dispersion at short distances.

### 2.2. Methane Emissions Technology Evaluation Center (METEC) Facility

**Figure 3:**
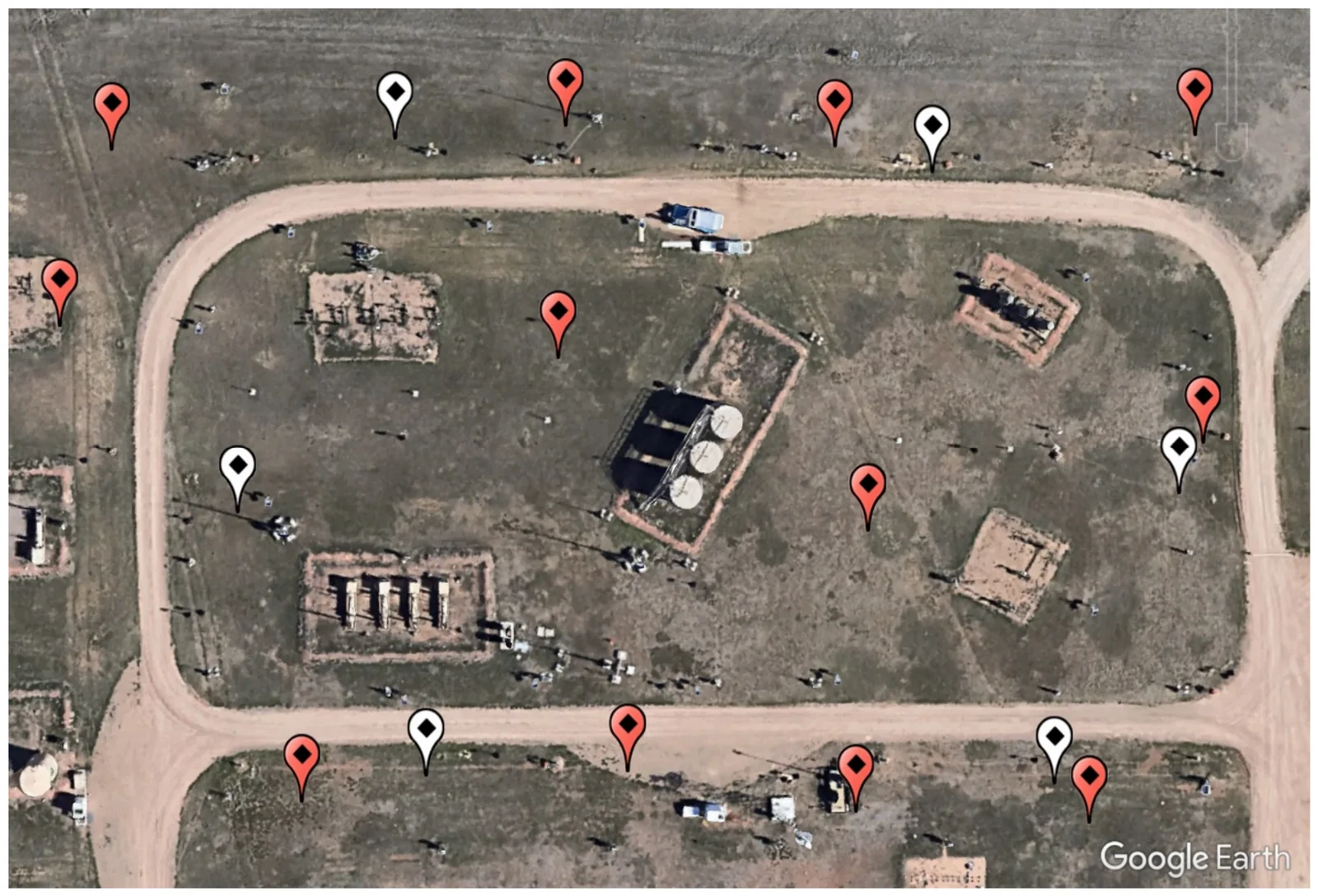
The Colorado State University METEC test facility, located in the northwest area of Fort Collins, Colorado. Placements are shown for BluBird 1.5 nodes during ADED 2022 (white symbols) and BluBird 2.0 nodes for ADED 2024 (red symbols) (image courtesy Google Earth; Imagery Date: 23 April 2023, lat 40.595782°, lon −105.139867°, elev 5142 ft., eye alt 5576 ft.).

During the 2022 and 2024 ADED programs, natural gas was released at the METEC facility (Figure 3) from any of five equipment groups, and from different equipment locations within the groups. Earthview deployed six BluBird 1.5 nodes for ADED 2022 (white symbols) and 12 BluBird 2.0 nodes (red symbols) for ADED 2024 at the positions indicated in Figure 3.

### 2.3. ADED 2022 and 2024 Testing Programs

**Figure 4:**
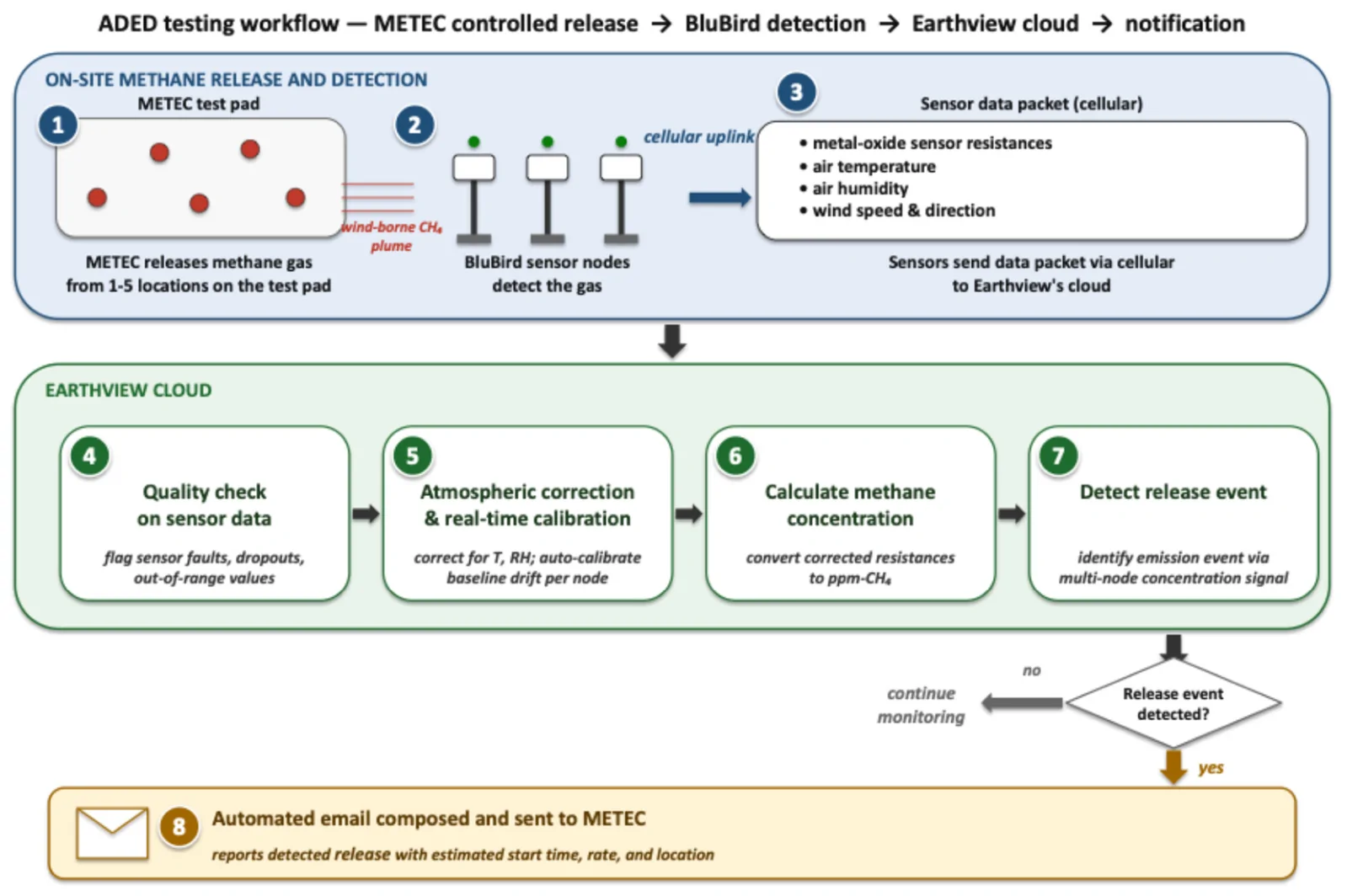
Process flow for BluBird data collection and analysis as part of the ADED 2024 program.

Details of the ADED protocol used for 2022 and 2024 and overviews of participants’ results are available at https://metec.colostate.edu/aded-testing-results/ (accessed on 16 June 2026) and are summarized by [18,19,20]. The overall flow of the ADED testing process is illustrated in Figure 4. (For reference, in the review of ADED 2024 provided by [20], the BluBird CEMS is “Participant C”). Test participants were required to report release detections, including start and end times, as well as additional required and optional information such as estimated release rate and source location, via email to METEC’s server. The participant-supplied detection information was parsed by METEC using criteria outlined in the protocols to classify each release as either successfully detected (“true positive”; TP), missed (“false negative”; FN), or as a detection report not associated with an actual release (“false positive”; FP).

The 1.5 version of the BluBird system was used in 2022, with BluBird 2.0 employed for ADED 2024. BluBird 2.0 includes hardware and software improvements compared to BluBird 1.5. For example, whereas the leak detection procedure used with the BluBird 1.5 system during ADED 2022 involved some manual interpretation, the detection procedure for BluBird 2.0 was fully automated. BluBird 2.0 also includes extensive upgrades for emission source location and quantification compared to BluBird 1.5. For ADED 2024. The time series of emission rates calculated from sensor node readings, reported at approximately 30-s intervals, were analyzed using a moving time window to identify likely emission events. Specifically, the emissions data were analyzed for 10-min segments within a moving 60-min window. If an emission rate within the 10-min window exceeded a pre-defined threshold, then that 10-min period was flagged. Methane concentration, along with wind speed and direction (measured at each node) during each 10-min time segment, was used to help identify the most likely emission source location using an automated procedure. Once 60 min had elapsed, the count of the number of 10-min segments that exceeded the emission threshold was compared to a second threshold. If enough 10-min segments were flagged, then the system concluded that an emissions event had occurred. The start time for the event was set as the time at the start of the 10-min increments. (This same basic process, with some enhancements, is currently used in the newer BluBird 2.5 system.). We note these details here since, as discussed below, this use of time segments and assignment of event start times affected the grading of BluBird’s leak detection performance as part of the ADED tests.

As implemented for the ADED 2024 testing, if the automated source location step used by BluBird 2.0 identified more than one potential source during an emissions event, each of these additional sources was considered a potential detection. After the initial detection report had been emailed to METEC, if the automated detection routine had not assigned a high confidence level to one or more sources, then these additional detections were reviewed manually. If any were considered likely to be true detections, the initial detection report was modified to include them and then updated via a follow-on email to METEC (such revisions were allowed by the ADED protocol for up to one week after the initial reporting).

**Figure 5:**
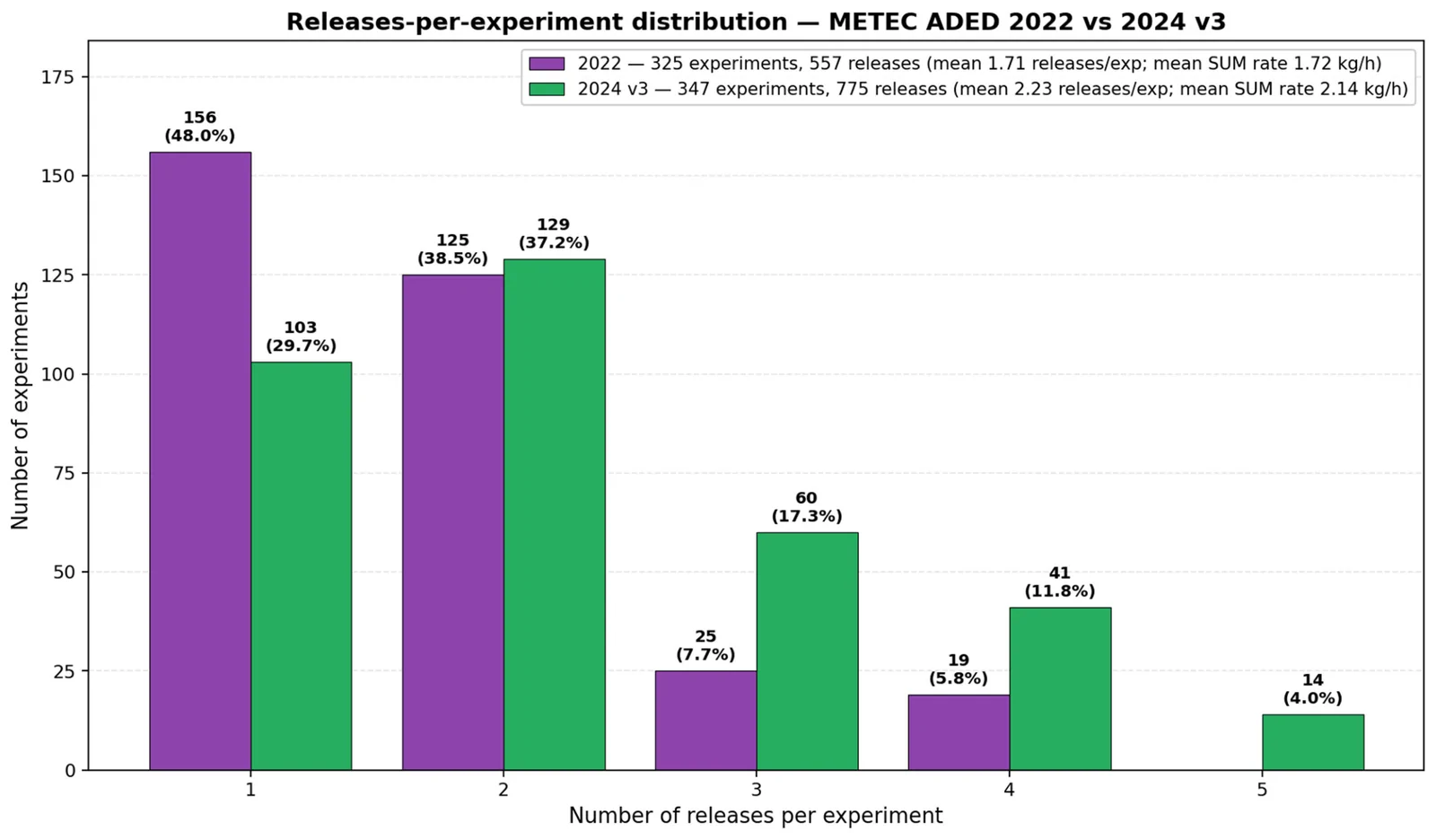
Number of natural gas releases per experiment for ADED 2022 and ADED 2024.

The ADED 2022 testing phase extended from 1 February to 11 May 2022, with a total of 325 release periods, (referred to as “experiments” by METEC), comprising 557 individual releases. The testing phase for ADED 2024 spanned 6 February–29 April 2024, with a total of 347 experiments made up of 775 separate releases (Figure 5). Each experiment consisted of from one to four simultaneous releases in 2022 and one to five releases in 2024, from any of the five “equipment group” locations on the test pad. Compared to ADED 2022, ADED 2024 included considerably more multiple-release experiments, fewer single-release experiments, and the addition of experiments that included five simultaneous releases. For ADED 2024, 73% of releases were at rates below 1.0 kg/h, with 34% of releases being less than 0.5 kg/h in 2022 and 40% of releases less than 0.5 kg/h in 2024. The most frequent rate range in 2022 was 0.5–1.0 kg/h (34% of releases), while in 2024 the most populated range was for rates < 0.5 kg/h, containing 33% of releases.

### 2.4. Analytical Methods

The methodology employed here addresses emission detection in terms of detection capability, time to detection and time to alerts; ability to accurately reproduce emission event timing and duration; emission quantification; and localization of the emission source. Probability of detection (PoD) at the 90% level as a function of emission release rate is estimated using maximum-likelihood logistic regression on the binary pairings of true positive/false negative outcomes for bins of rate for univariate estimation, and for rate, duration, and wind speed for trivariate estimation. We also calculated PoD using an exponential fit for direct comparison with PoD curves given by [19].

In addition to the detection classification results and direct comparisons of actual and reported release emission rates, we calculate total gas emissions per experiment, which we define as the sum of the emission rate for each release multiplied by the release duration. The totals are summed over different time intervals ranging from daily to the full ADED test periods.

To help diagnose possible reasons for classification error, we use time series of BluBird-derived methane concentrations, as presented on Earthview’s data dashboard (dashboard.earthview.io).

The ADED results data set used here, including the initial version 3 ADED 2024 data supplied by METEC along with the additional derived groupings (single-release, per-experiment, alternative classification criteria listed in Section 2.4.2), are provided in the Supplementary Materials. Five BluBird detection reports in the ADED 2024 master results file (Source IDs 284, 352, 353, 706 and 804) had been entered with release end times that were earlier than the start times and off by exactly 24 h. Since these reports appear to be typographical errors that otherwise would be valid detections, the dates were corrected in the results data file used here.

Anthropic Claude (versions Sonnet 4 and Opus 4) was used for statistical analyses (probability of detection, correlation analyses, basic statistical summaries), along with graphics generation (all figures except Figure 1, Figure 2 and Figure 3). Results were verified by comparing AI-compiled summaries to calculations performed manually (for example, using separate spreadsheet calculations) as well as by applying standard cautions for AI use, such as routinely “re-asking” Claude to carry out tasks and then checking for consistency.

#### 2.4.1. Meteorological Data

To investigate possible relationships between atmospheric conditions and emission detection and quantification, we use the wind information provided within the METEC ADED data reports along with wind speed and direction reported by each BluBird node. Surface boundary layer conditions are assessed using NOAA High-Resolution Rapid Refresh (HRRR) model fields [48] obtained from the NOAA Open Data archive on Amazon Web Services S3 (https://noaa-hrrr-bdp-pds.s3.amazonaws.com/, accessed on 16 June 2026), using the Herbie Python package (https://github.com/blaylockbk/Herbie, accessed on 16 June 2026; version 2026.3.0).

#### 2.4.2. Detection Overlap Accuracy and Alternative Data Groupings

**Figure 6:**
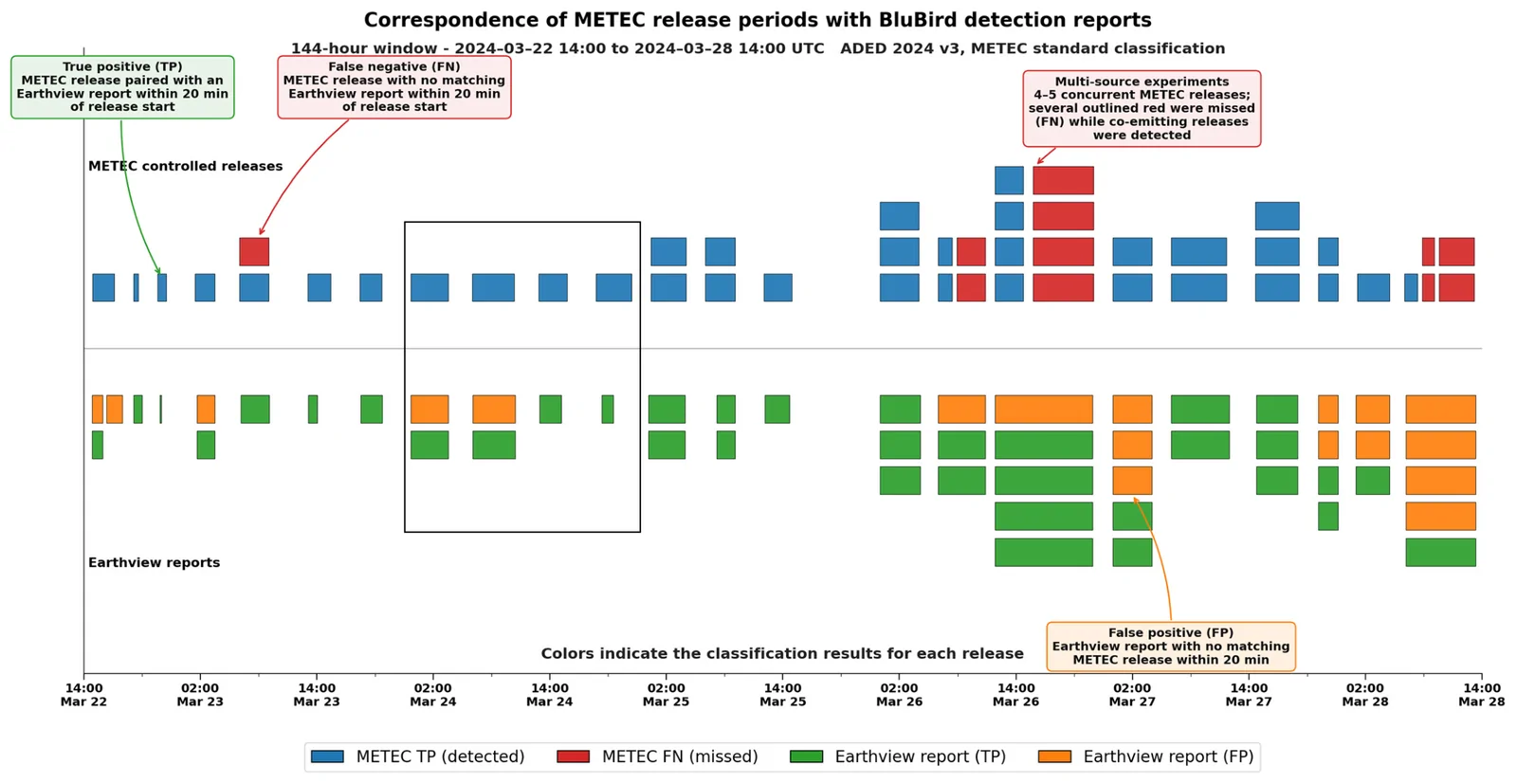
Comparison of ADED release periods (top half of the graph) with BluBird-reported release detections (bottom half of the graph) for the period 23 March–28 March 2024 (UTC). Each stack of bars in the graph’s top half represents one ADED experiment. The blue and red colors indicate whether the release was considered detected (blue) or missed and classified as a false negative (red). The green and orange colors indicate whether the BluBird report was graded as a detection and classified as a true positive (green) or as a report that had no corresponding METEC release (orange). Multiple bars in a stack indicate multiple releases during a single experiment. BluBird-derived methane concentrations for the area in the black box are presented below in Figure 7.

A simple but effective way to see how well ADED releases were detected is to plot timelines of releases and BluBird reports. This approach (Figure 6) reveals important aspects of CEMS capabilities, such as overall detection, the accuracy of start and end times (i.e., release duration), the ability to detect short releases, and the ability to distinguish between individual releases separated by short time gaps. It also illustrates clearly how the above-mentioned use of multiple releases and METEC’s start-time grading criterion affects classification results.

**Figure 7:**
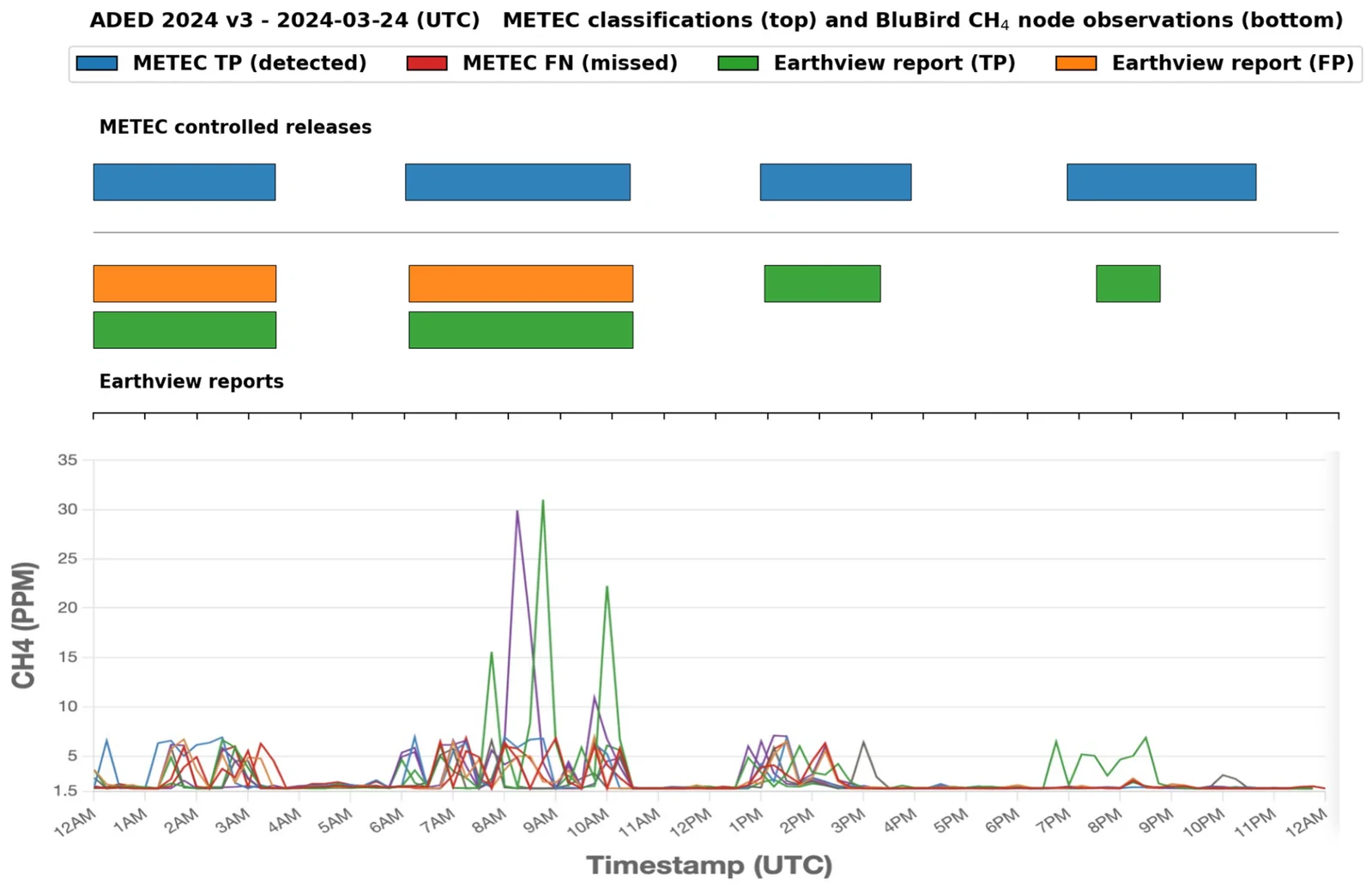
Time series of BluBird-reported methane concentrations (line graph), corresponding METEC releases (blue bars in the top half of the top panel), and BluBird detection reports (green and orange bars in the bottom half of the top panel) for 24 March 2024 (the subset highlighted by the black box in Figure 4). The color scheme for the bars is as in Figure 6. Each of the lines in the line graph represents concentrations estimated by for an individual BluBird node, with each node’s data shown in a different color.

In Figure 6, ADED releases (top half of the graph) and BluBird detection reports (bottom half of the graph) are displayed for a randomly selected, representative 144-h period from 23 March through 28 March 2024 (additional plots at 144-h intervals spanning the full test period are included in the Supplementary Materials). The colors show the results of METEC’s standard grading for true positives (TP; blue and green), false negatives (FN; red), and false positives (FP; orange). Each stack of bars in the graph’s top half represents one ADED experiment. Multiple bars in a stack indicate multiple releases during a single experiment. Interpretation of these overlay comparison plots is presented in Section 3.1.

Figure 7 is an example of the underlying data used by BluBird to detect release periods. It shows a typical example of BluBird-reported methane concentrations and corresponding detection reports for four experiments spanning a 24-h period. During the METEC releases during this time period, estimated concentrations ranged from around five to 30 ppm, settling at around 1.9 ppm between releases. For this set, the ADED grading resulted in four TPs and two FPs, with the latter due to BluBird’s overestimate of the number of simultaneous releases during the first two experiments. The typical correspondence seen between the measured methane concentrations, the BluBird-reported detection times, and METEC’s actual release periods seen in Figure 7 further supports the conclusion that overlap percentage is a worthwhile measure of release detection.

#### 2.4.3. Alternative Grading Using Site Detection, Relaxed Start-Time Threshold, and Detection Overlap

As noted above, a key objective of this study is to describe the CEMS performance in terms of the ability to detect that at least one leak is present on a site. This is in contrast to previous reports on ADED test results, which fail to differentiate between this and the detection of multiple, simultaneous releases. To address this, we divide METEC’s supplied version 3 report into three sets: (1) the full original data set consisting of all releases for all experiments and which follows the standard METEC approach to date (referred to as “all releases”, i.e., single-release and multiple-release experiments); (2) a “per experiment” data set that represents active-site leak detection (i.e., detection of at least one release during an experiment); and (3) a data set consisting of experiments with only one release per experiment (“single releases”). By dividing up the analysis in this manner, we can duplicate the METEC-reported results using the “all-release” subset, consistent with [20,21,22]. We can also investigate BluBird’s performance for active-site leak detection and quantification using the “per experiment” data set, and can separate out the effects of multiple releases using the “single release” data set. For the per-experiment set, an experiment is assigned a true positive (TP) score if BluBird had been assigned at least TP during the experiment. Experiments that have no corresponding BluBird detection report are assigned as false negatives (FN). A detection report that does not correspond to an experiment period is assigned a false positive (FP).

As discussed in Section 3.1 below, there are many cases (about 15% of the total number of detection reports) where the BluBird reports appear to correspond quite closely with the METEC release times but which are classified as FNs and/or FPs under METEC’s standard grading. This does not imply that the reports are misclassified. Instead, such reports likely relate to actual releases but did not meet METEC’s criteria. Many of these are due to reported times being slightly earlier than the METEC start times. The method used by Earthview to automatically assign leak-detection start times (Section 2.1) has the potential to yield a start time as much as 10 min earlier than the METEC-recorded start times solely due to book-keeping. Given this, we chose to also investigate the sensitivity of the overall BluBird performance assessment to alternative classification criteria that treat such reports as detections. We refer to these as potential detections to clarify that any improvements associated with inclusion of these additional detections are due to relaxing the classification criteria rather than a direct improvement in system performance.

Based on the graphical and time-offset evidence that these close matches to the METEC releases do in fact correspond to ADED release times, we devised two additional classification criteria to complement METEC’s standard ≤ 20.0-min start time threshold (“METEC standard”): (1) an early-start-time threshold of ≤30.0 min (“30-min”), and (2) a criterion that considers how well a BluBird detection report overlaps with a corresponding METEC release period. For the latter, the release and report need to overlap by at least 75% to be treated as a TP detection (“75% overlap”). Specifically, the overlap criterion is the percent of the METEC release period in seconds that is spanned by the BluBird detection period, or,

(1) $$percent\;overlap=\frac{\Delta t_{ov}}{T_{M}}\times 100$$

where

(2) $$\Delta t_{ov}=max\left(0,\;min(t_{M,end},\;t_{B,end})-max(t_{M,start},\;t_{B,start})\right)$$

and the symbols are defined as:

- $\Delta t_{ov}$ = duration of temporal overlap between the METEC release and the BluBird detection report, in seconds;
- $T_{M}$ = duration of the METEC release, in seconds;
- $t_{M,start},\;t_{M,end}$ = start and end times of the METEC release;
- $t_{B,start},\;t_{B,end}$ = start and end times of the BluBird detection report.

To qualify as a TP detection, the percent overlap must be ≥75, meaning that the Earthview emission report window must cover at least three quarters of the METEC release window.

Table 2 summarizes the classification criteria used for analysis. Each subset provides different insights into the BluBird CEMS performance. For reasons noted later, the ADED 2022 results are not sensitive to the alternative classification criteria, so only the original METEC standard classification is discussed. Using these classification criteria, separate subsets were made for the “all-release”, “per-experiment” and “single-release” data groupings. These additional classification criteria should be treated as secondary to the baseline METEC standard-classification results. They are presented here primarily as a way of quantifying the sensitivity of the overall results to relatively slight differences in how a successful detection is defined.

## 3. Results

### 3.1. Release vs. Detection Report Overlap

As introduced in Section 2.4.2, plots of ADED release times stacked on top of BluBird detection reports convey details regarding the correspondence of start and end times, the ability to detect small-duration releases and short gaps between releases, and a comparison of release rates versus reported rates. All the time periods discussed below have been randomly selected and are representative of the full ADED 2024 test period.

#### 3.1.1. Overlap Patterns, Gap Delineation, and Multiple Releases

**Figure 8:**
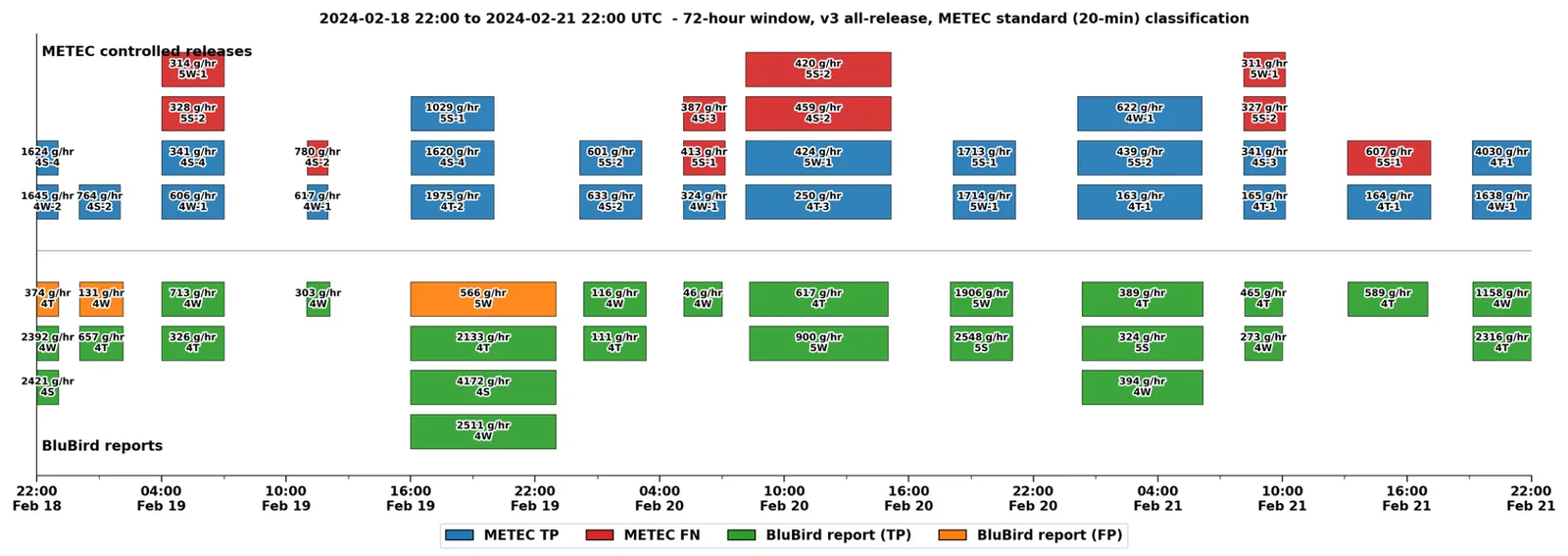
Comparison of ADED release periods (top half of the graph) with BluBird-reported release detections as assigned by METEC’s standard grading (bottom half of the graph) for the period 19–21 February 2024 (UTC). The organization and color coding are as in Figure 6. The labels on each bar are METEC’s reported gas release rates (g/h) or, for the BluBird reports, the estimated emission rates.

The time period in Figure 8 includes 13 individual experiments (each separate set of vertically stacked bars) consisting of a total of 34 releases. The organization and color coding are the same as the ones used earlier in Figure 6.

Several key points are immediately apparent in this figure and apply to all the subsequent interpretations of BluBird performance. (As noted earlier, a full set of overlay plots spanning the entire ADED 2024 period is provided in the Supplementary Materials). Based on the visible correspondence of detections with releases, it appears that BluBird was able to reliably map individual releases of different durations and to identify gaps between releases, including short gaps. Beyond the general detection patterns, the implications of multiple releases per experiment (the individual stacks of bars) are clear. For example, BluBird detected the presence of gas during all 13 experiments in the time period (or 100% detection, in terms of detecting at least one release during experiments). A total of 10 FNs were assigned for this period. Each of these FNs is a result of not reporting one or more simultaneous releases during individual experiments. In contrast, all the FPs are due to over-reporting the number of releases during multiple-release experiments. None of the FPs are due to reporting gas when none was present. The main takeaway is that BluBird detected all the cases during this period when at least one leak was present and would therefore have correctly alerted a client to consider a site inspection, and no FPs were generated that would have triggered an unwarranted site inspection.

**Figure 9:**
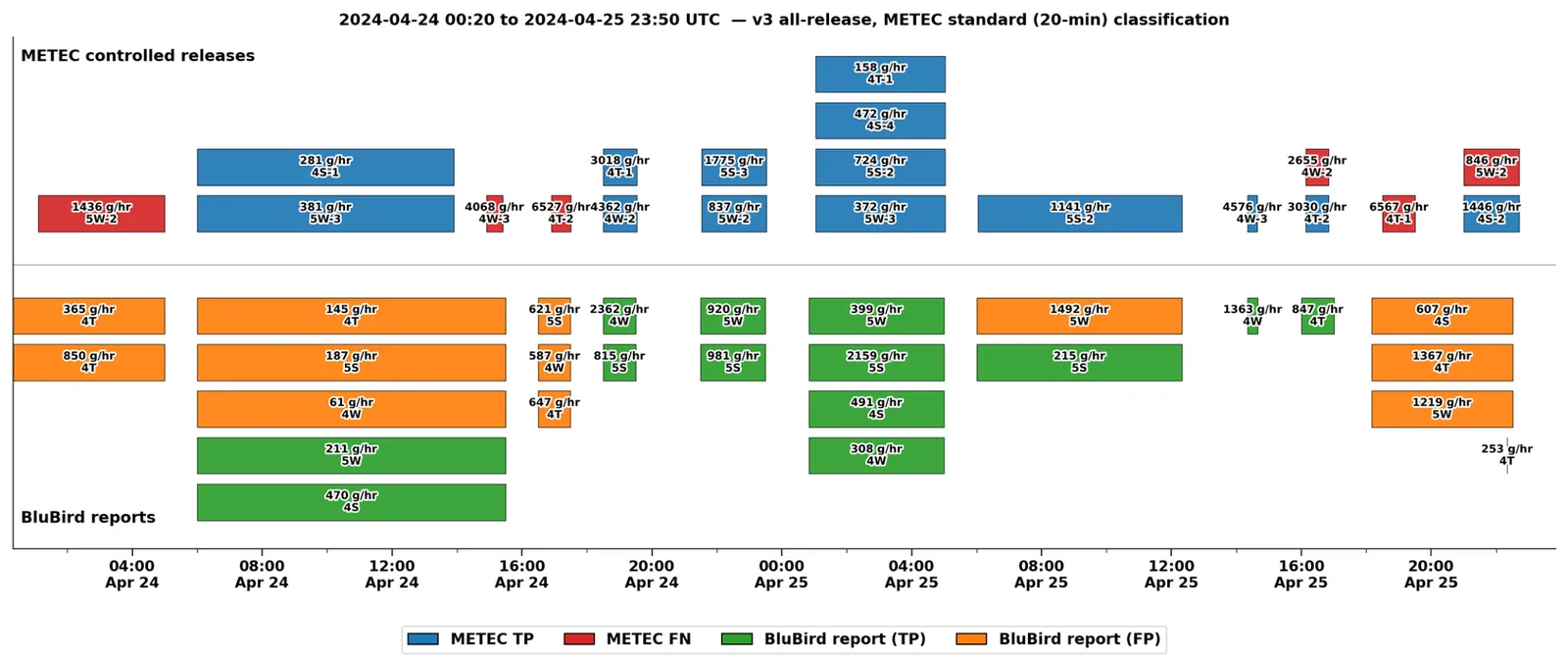
Comparison of ADED release periods with BluBird-reported release detections as assigned by METEC’s standard grading for the period 24–26 April 2024.

**Figure 10:**
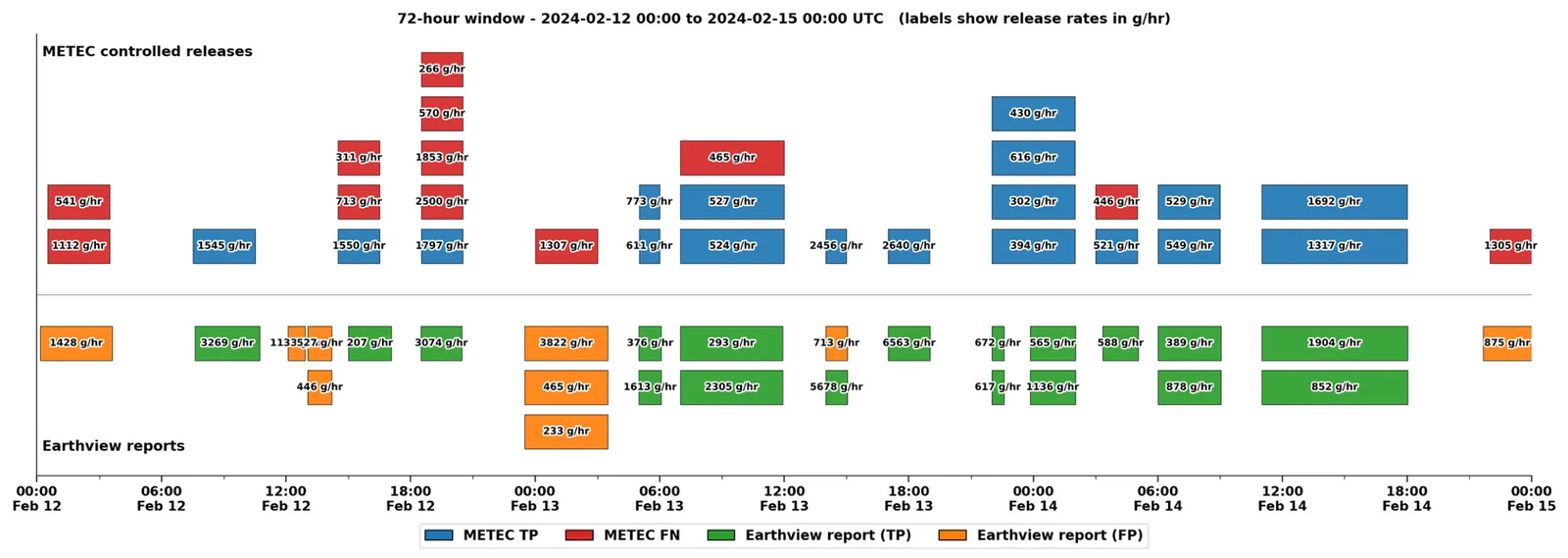
Overlap time series for the period 12–15 February, showing examples of report rejections due to start times less than one minute earlier than METEC’s allowable start-time threshold.

The experiments mapped in Figure 9 and Figure 10 below highlight the challenges posed by the ADED protocol, which included short-duration releases with short time gaps between releases. In this case, which is typical of the full data set, all the experiments were detected by the BluBird CEMS. Several relatively short releases were identified, as were some short time gaps between releases. However, the number of releases per experiment tended to be over-estimated. For the first experiment in this time series, BluBird was assigned two FPs (the orange bars) and an FN (the red bar) due to reports having start times that were too early. Three additional FPs were assigned for the second experiment due to over-reporting detections, along with an FN for failing to assign the short release as a separate event. BluBird detected the short experiment at around 17:00 h on 24 April (experiment number four in this series), but the three reported detections were again assigned as FPs (along with one FN) due to a reported start time that was 24.2 min earlier than METEC’s listed start time. For this set of experiments, application of the alternative 30-min start time threshold re-classifies experiment number four as one TP, two FPs and zero FNs instead of three FPs and one FN. The start time differential for the other two experiments is greater than 30 min, but they qualify as potential detections using the 75% overlap classification criterion. In Figure 7, the BluBird reports for the first and last experiments were again rejected due to having start times slightly earlier than METEC’s 20-min threshold. The fifth experiment, for example, has a reported start time of 30.1 min earlier than the METEC release start time. We acknowledge that, although there is strong visual alignment between the BluBird reports and the METEC release times for cases where the BluBird detections were rejected (as seen in the figures here and the full set of overlap plots included in the Supplementary Materials), we have no independent data to further support the conclusion that these are potential detections.

For all detection reports where BluBird assumed a continuous release when a gap was in fact present, such as between experiments two and three and between experiments 11 and 12 at around 18:00 UTC in Figure 9, BluBird-reported methane concentrations were above background for at least one node. The FPs assigned during 12:00–14:00 on 12 February in Figure 10 are two out of six cases during ADED 2024 where BluBird reports had no overlap with a METEC release (e.g., “true” FPs). In five of these cases, at least one sensor node was reporting concentrations above background. The possible implications of this are analyzed further in Section 3.9.

#### 3.1.2. Accuracy of Detection Report Overlap Periods

To help quantify the visual evidence from the overlay plots that some BluBird reports represent potential detections, we calculated a Jaccard Index (or Jaccard Similarity Coefficient; [49]), which describes how well two sets intersect. A Jaccard Index value of 0.0 indicates a complete mismatch and 1.0 indicates a perfect match. In our case, the sets are the ADED 2024 release times and the corresponding BluBird detection report times; a high degree of intersection suggests that the two series are substantially similar and therefore could reasonably be treated as likely potential detections. The overall index is 0.92, with an interquartile range (IQR) of 0.77–0.98 encompassing the middle 50% of the cases. This is consistent with the overlap plots presented earlier in suggesting that the BluBird reports correspond well to the METEC reports. Lower similarity values are almost entirely due to early start times rather than mismatched ending times. With just a few exceptions, these early-start cases otherwise overlap well with a corresponding METEC release. It is worth reiterating that these are not METEC classification errors, but rather detections that could reasonably be considered as corresponding to a METEC gas release.

### 3.2. Detection Performance

As noted earlier in Section 2.4.3, to be able to assess BluBird performance in terms of active-site leak detection independent of multiple simultaneous releases, we divided the ADED 2022 and 2024 data sets into three sets: (1) the full set of results consisting of all releases for all single-release and multiple-release experiments (the “all-release” data set, which is the same data set reported on in [19,20]; (2) a “per-experiment” data set, where an experiment is defined as a period with at least one METEC release; and (3) a subset consisting only of experiments with just one release (“single-release” experiments).

The total numbers of METEC’s assigned TP, FN, and FPs for ADED 2022 and ADED 2024, along with results for the data subsets and alternative classification criteria, are reported in Table 3, Table 4, Table 5 and Table 6. “Rescues” represent detection reports that were classified as FPs using METEC’s standard grading, but which transitioned to potential TPs when the alternative classification criteria (30-min start time threshold or 75% overlap) were applied.

Overall detection rates for experiments improved from 57% for ADED 2022 to 91% for ADED 2024 using METEC standard grading. A key aspect is that, for ADED 2024, detection success (TP% in Table 3, Table 4, Table 5 and Table 6) is considerably different depending on whether the measure is detection of individual experiments (i.e., at least one release per experiment) or detection of all releases within experiments (90.5% versus 69.2%). The BluBird FNs and FPs in the 2024 results are primarily due to under- or over-reporting of releases during multiple-release experiments or to slight report-timing offsets rather than to missed or over-reported experiments. Misclassifications during multi-release experiments account for 94% of FNs in the ADED 2024 results, and slightly fewer (82%) for ADED 2022. This is consistent with the patterns seen in the overlay plots in Section 3.1.

**Figure 11:**
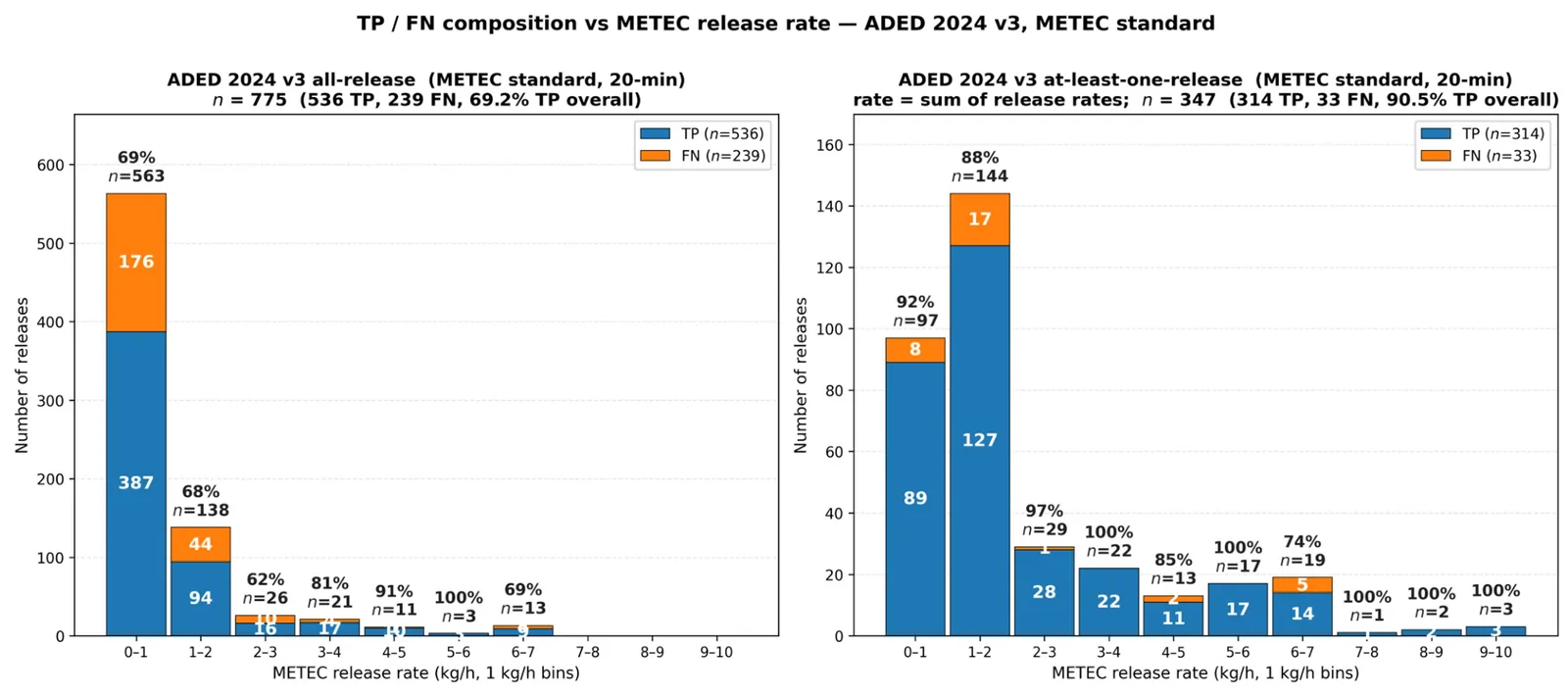
Detection percentage versus emission release rate bins for ADED 2024 using the standard METEC grading (20-min start-time threshold). All-release results (**left panel**) and per-experiment (at least one release detected) results (**right panel**).

**Figure 12:**
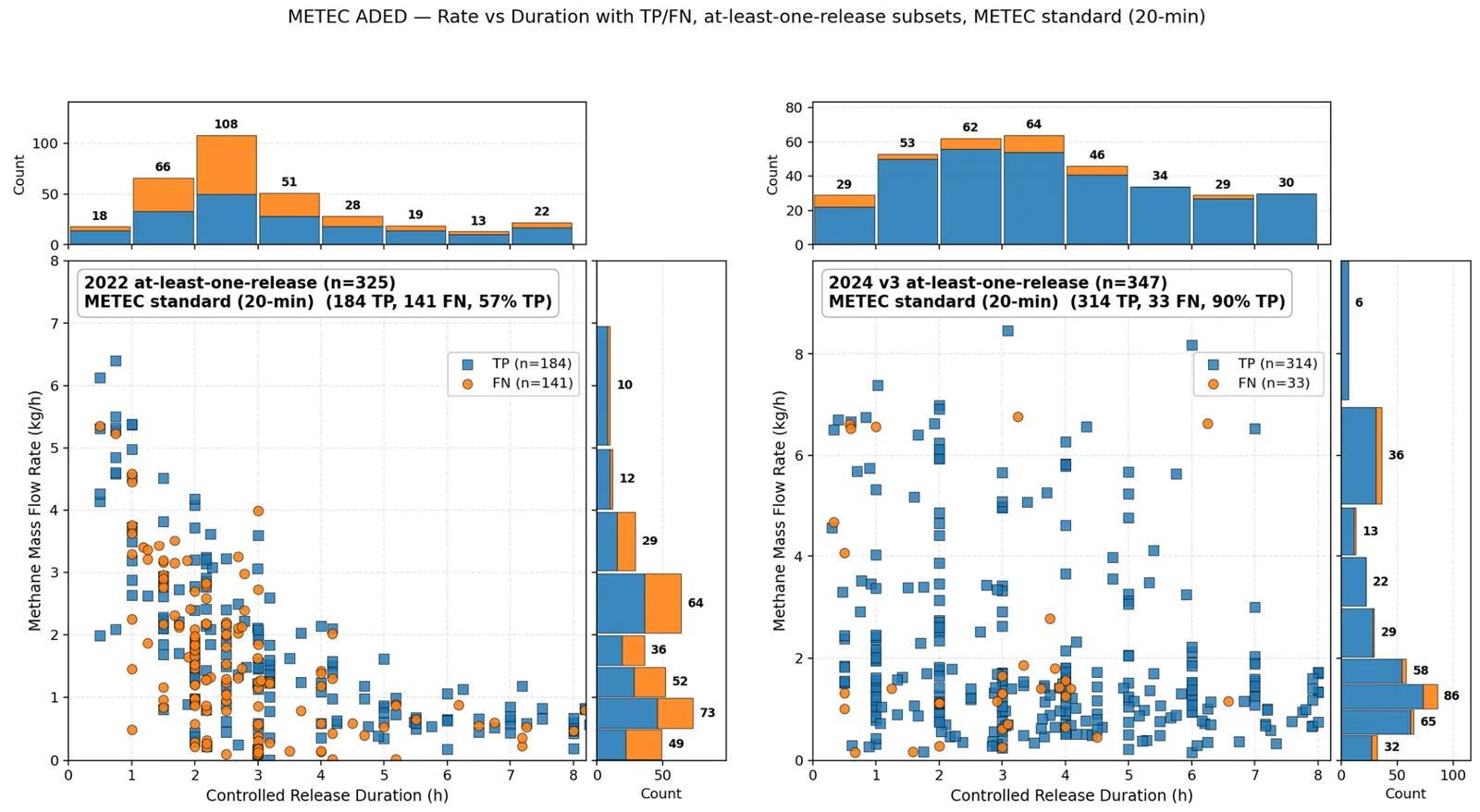
True positive (TP) and false negative (FN) assignments plotted as a function of release duration (hours) and methane release rate (kg/h) for the ADED 2022 (**left panel**) and ADED 2024 (**right panel**) per-experiment subsets. The adjacent histograms show TP and FN counts for duration and release rates.

**Figure 13:**
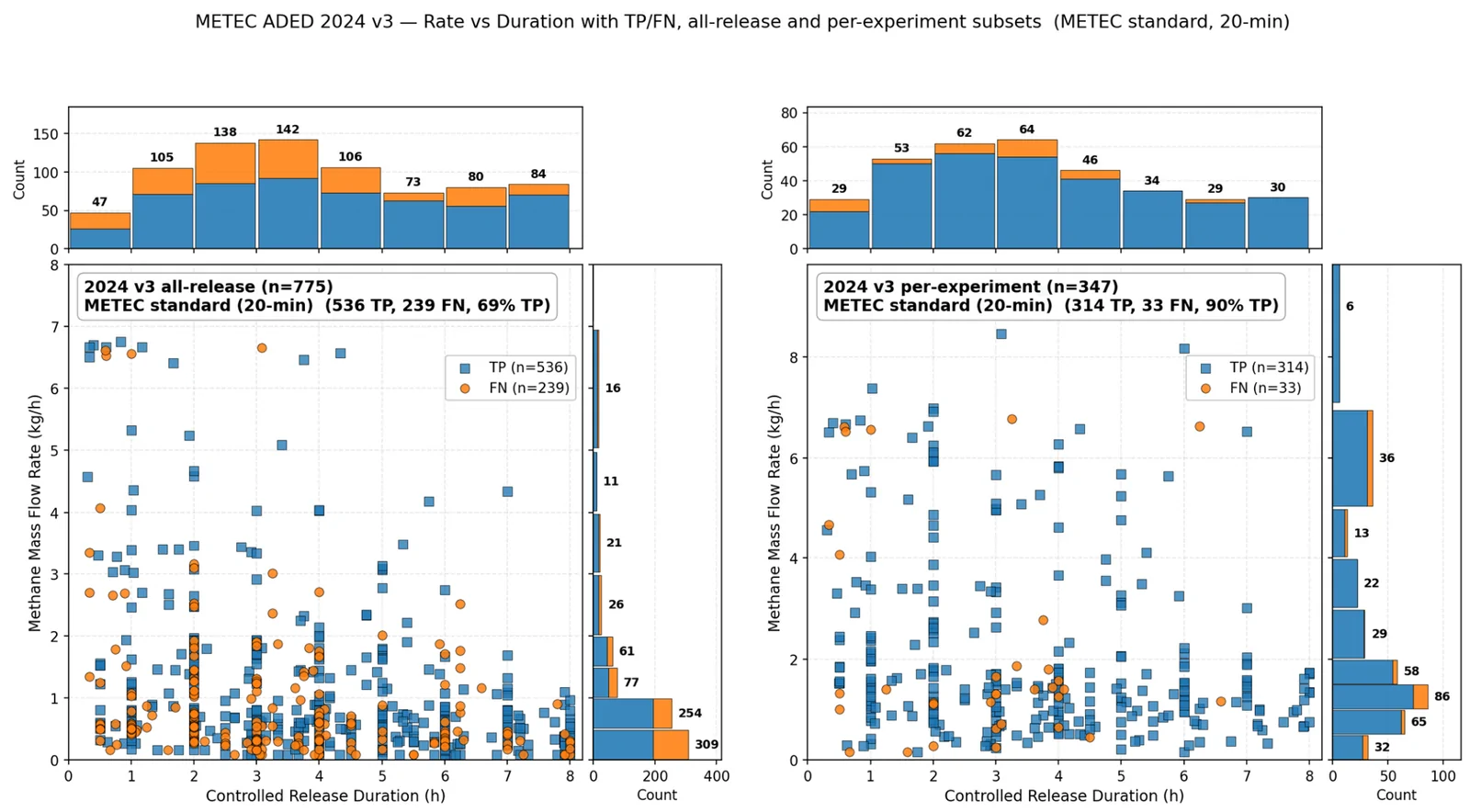
True positive (TP) and false negative (FN) assignments plotted for release duration (hours) and methane release rate (kg/h) for the ADED 2024 all-releases subset (**left panel**) and per-experiment subset (**right panel**). The adjacent histograms show the TP and FN counts for the respective x and y axes.

**Figure 14:**
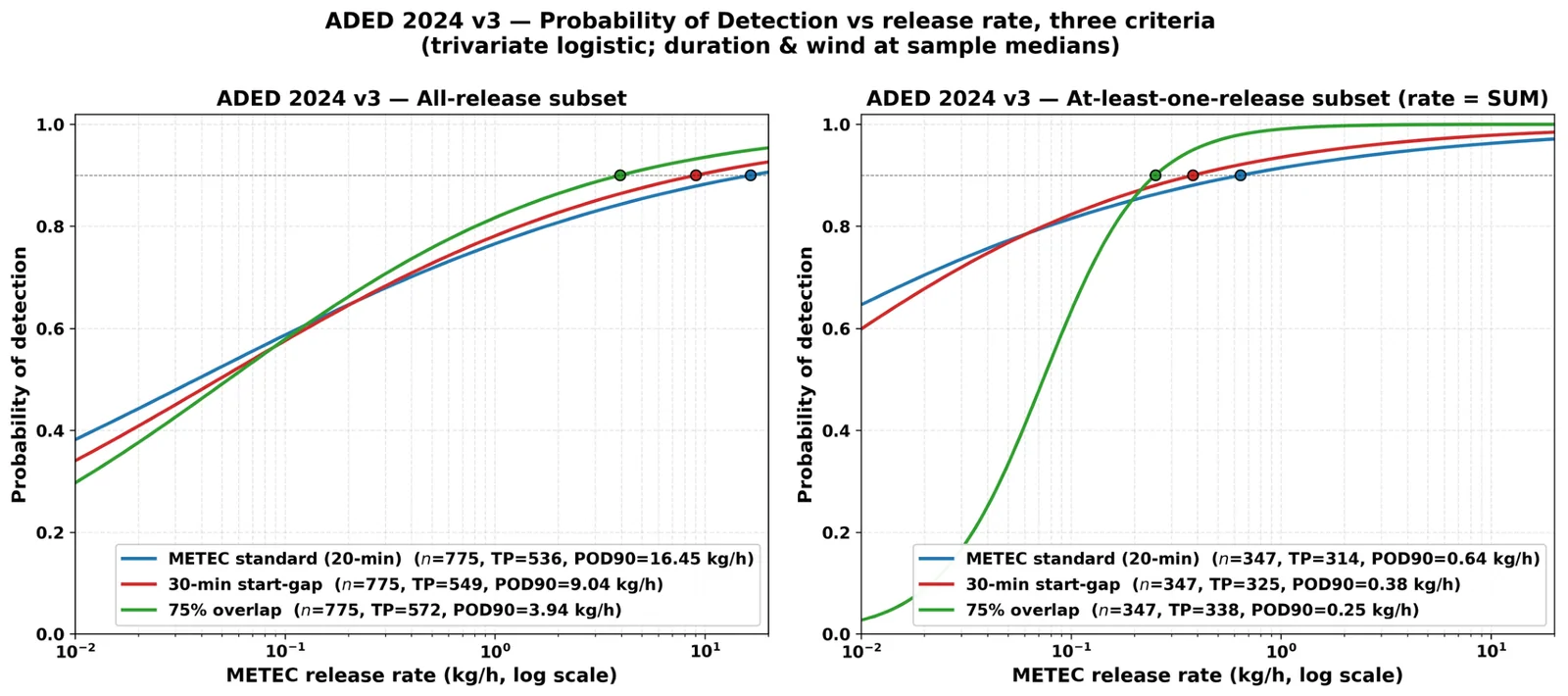
BluBird PoD as a function of release rate for the per-experiment subset, for which any release detected results in a detected experiment (i.e., a TP). Curves show the multivariate logistic fit under for the METEC standard classification. Curves obtained using the alternative 30-min and 75% overlap criteria are included for comparison. Ninety percent PoD release-rate values are marked at each curve’s intersection with the “PoD = 0.90” reference line. Note that the x-axis is on a log scale. Note that the 30-min start gap and 75% overlap indicators pertain to the potential detections added under these alternative classification criteria and are not formal ADED measures. The colored circles indicate the release rates where each data set intersects the 90% PoD axis.

**Figure 15:**
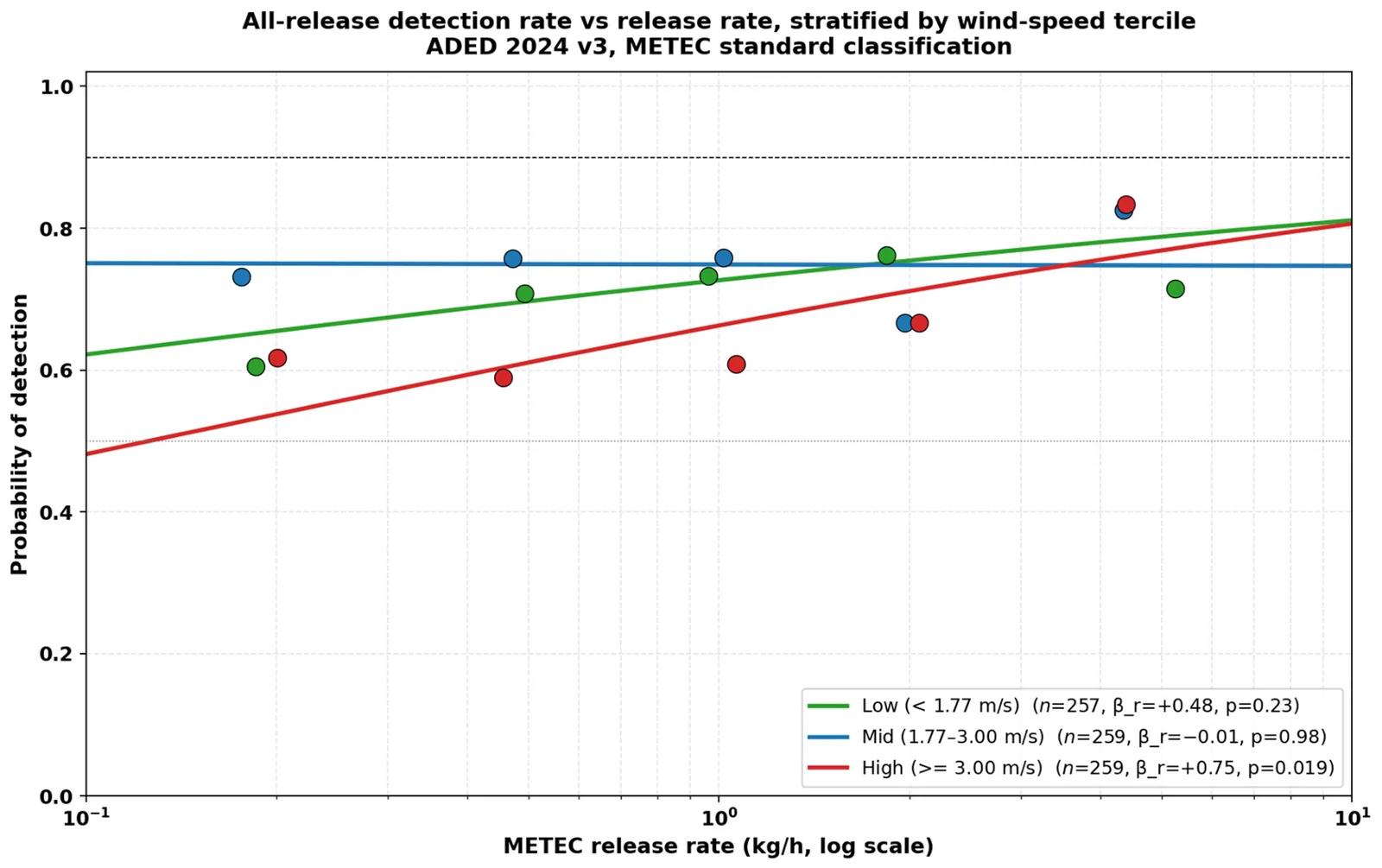
Relationship between release rate and probability of release detection for different ranges of wind speed. Colored circles are empirical detection rates for each release-rate bin within a wind-speed category, plotted at the bin’s mean release rate; only bins with ≥5 releases are shown.

The detection percentages for ADED 2024 as a function of methane release-rate bins are summarized in Figure 11, Figure 12, Figure 13, Figure 14 and Figure 15. Comparing the all-release versus the per-experiment results again highlights the significance of considering the ADED performance in terms of per-experiment (site-level) leak detection (Figure 9 and Figure 10).

Improvement in detection rates between ADED 2022 and ADED 2024 is apparent across all release rate bins, with detection improving by roughly a factor of 2. Results are similar between the per-experiment set (Figure 11) and the all-release set.

Release rate and release duration (Figure 12) can both be expected to affect detection rates for fixed-position point sensors such as the BluBird CEMS. This was the case for the ADED 2022 BluBird 1.5 results; detection rates correlate significantly with higher release rates as well as with longer release duration. For ADED 2024, though, no statistically significant relationship with release rate is found; since detection rates are above 90% across the release-rate range, there is little variability to be accounted for. However, release duration was a significant factor in detection rate for both ADED programs.

Figure 13 compares detection versus release rate and duration for the ADED 2024 all-releases and per-experiment sets. The fact that many more FNs appear in the all-releases plot than in the per-experiment plot shows that, as discussed earlier, most of the FNs are missed releases in multiple-release experiments rather than failure to detect entire experiments.

### 3.3. Probability of Detection

It is useful to define what “probability of detection” means in the context used here, as estimated from the ADED testing. It represents the probability of detecting a single, continuous release with a duration of between roughly 20 min and several hours (the durations seen during ADED), under weather conditions typical of February–April in northern Colorado. Statistically derived probabilities of detection as functions of gas release rate, release duration and wind speed were calculated using logistic regression with trivariate analysis, which takes into account that the detection rate may depend simultaneously on all three parameters. The ADED 2024 and ADED 2022 per-experiment results are presented in Table 7 and Table 8 and Figure 14, calculated for release rate at median release duration and median wind speed (i.e., trivariate-at-median results). For the per-experiment subset, the release used is the sum of the rates for all releases during an experiment.

A key result is the very large difference between 90% PoD estimated using the all-release data set (the full data set where releases are treated independently) and the per-experiment data sets that treat experiments as individual site-level events. It should be recalled that the all-release data set does not differentiate whether TPs and FNs are associated with site-level leak detection or detection of individual releases within simultaneous-release experiments. For this data set, a TP or FN is assigned for every release. In contrast, for the per-experiment data set, a TP or FN is assigned to each experiment; therefore, if at least one release during an experiment is detected, that experiment is assigned a TP. The all-release data set combined with the standard METEC start-time threshold is equivalent to the data sets analyzed by METEC investigators in [18,19,20].

For the per-experiment set using the standard METEC grading, the calculated 90% PoD release rate for detecting whether a leak is present on a site improves to 0.64 kg/h in the ADED 2024 results, compared to 16.44 kg/h using the all-release set; a projected value that lies beyond the range of release rates used during the ADED program and therefore is not physically relevant. A critical choice in presenting and interpreting the ADED results is therefore whether to define PoD only in terms of detecting all releases during multiple-release experiments as in [18,19,20] or to also consider the ability to detect any release during an experiment (i.e., an active-site detection). 

The PoD estimation further illustrates relationships between detection rates and other parameters. Correlations of release rate, duration and wind speed with detection are weak for the ranges encompassed by the ADED 2024 test period. Combined, the three predictors together account for a small fraction of the variance in per-experiment detection outcomes (from 2% to 7% depending on the correlation measure used). This is consistent with the near-90% detection rate, which leaves little variability to explain. However, when considered individually, release duration is the strongest predictor of detection (logistic β = +1.15, *p* = 0.026; Spearman ρ = +0.118, *p* = 0.028; McFadden R^2^ = 2.2%), roughly twice as strong as wind speed by pseudo-R^2^ (McFadden R^2^ = 1.2%, *p* = 0.11) and roughly eight times as strong as release rate (McFadden R^2^ = 0.27%, *p* = 0.45).

**Figure 16:**
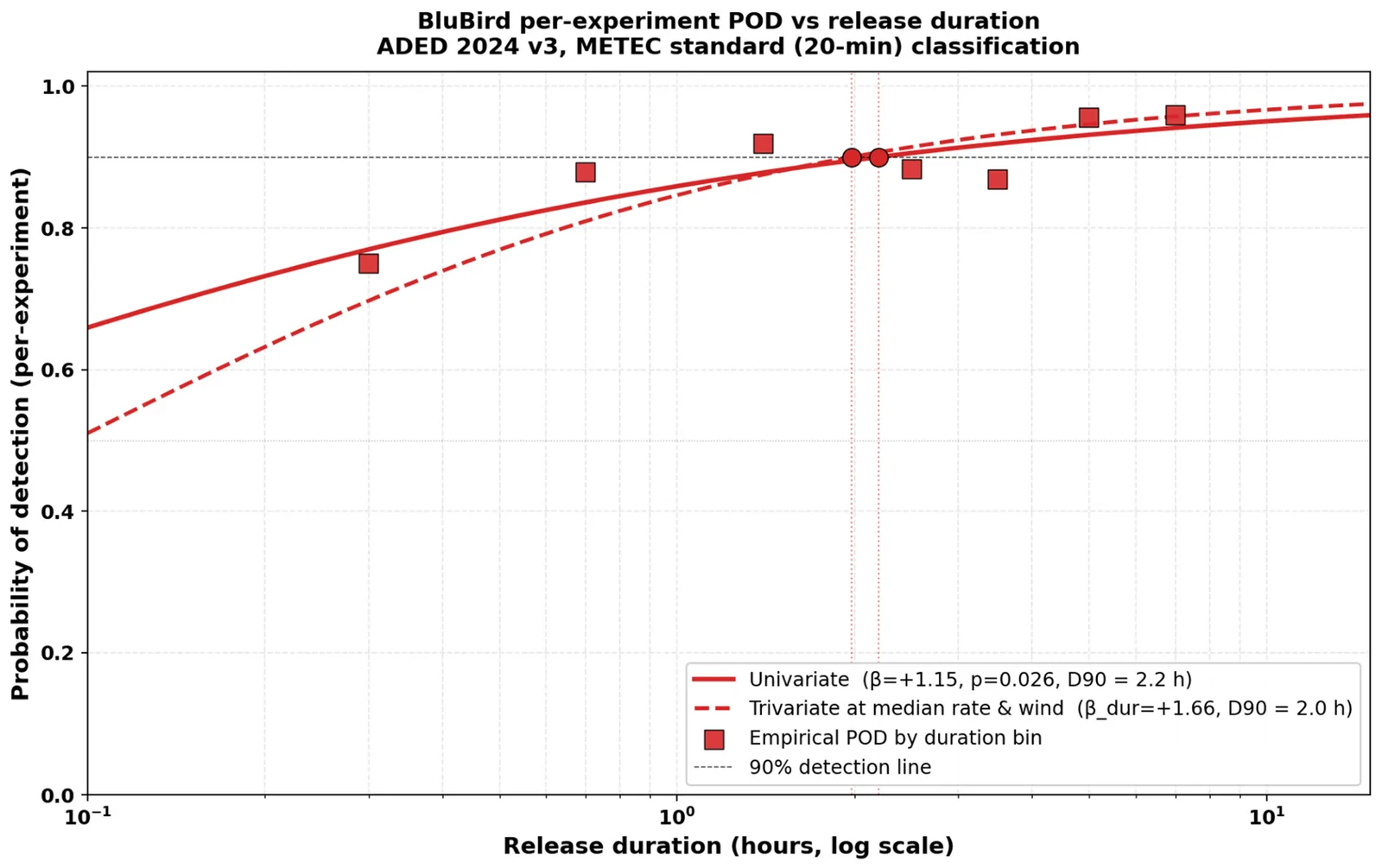
BluBird PoD as a function of release duration for the per-experiment subset as calculated individually (solid line) and with release rate and wind speed as additional predictors (the trivariate dashed line), calculated for their median values, to show the effect of duration alone after accounting for the other two variables.

When stratified by wind speed, correlation with release rate becomes more apparent (Figure 15). In terms of PoD as a function of release duration (Figure 16), the data suggest a 90% likelihood of detecting a release that is at least 2.2 h long.

**Figure 17:**
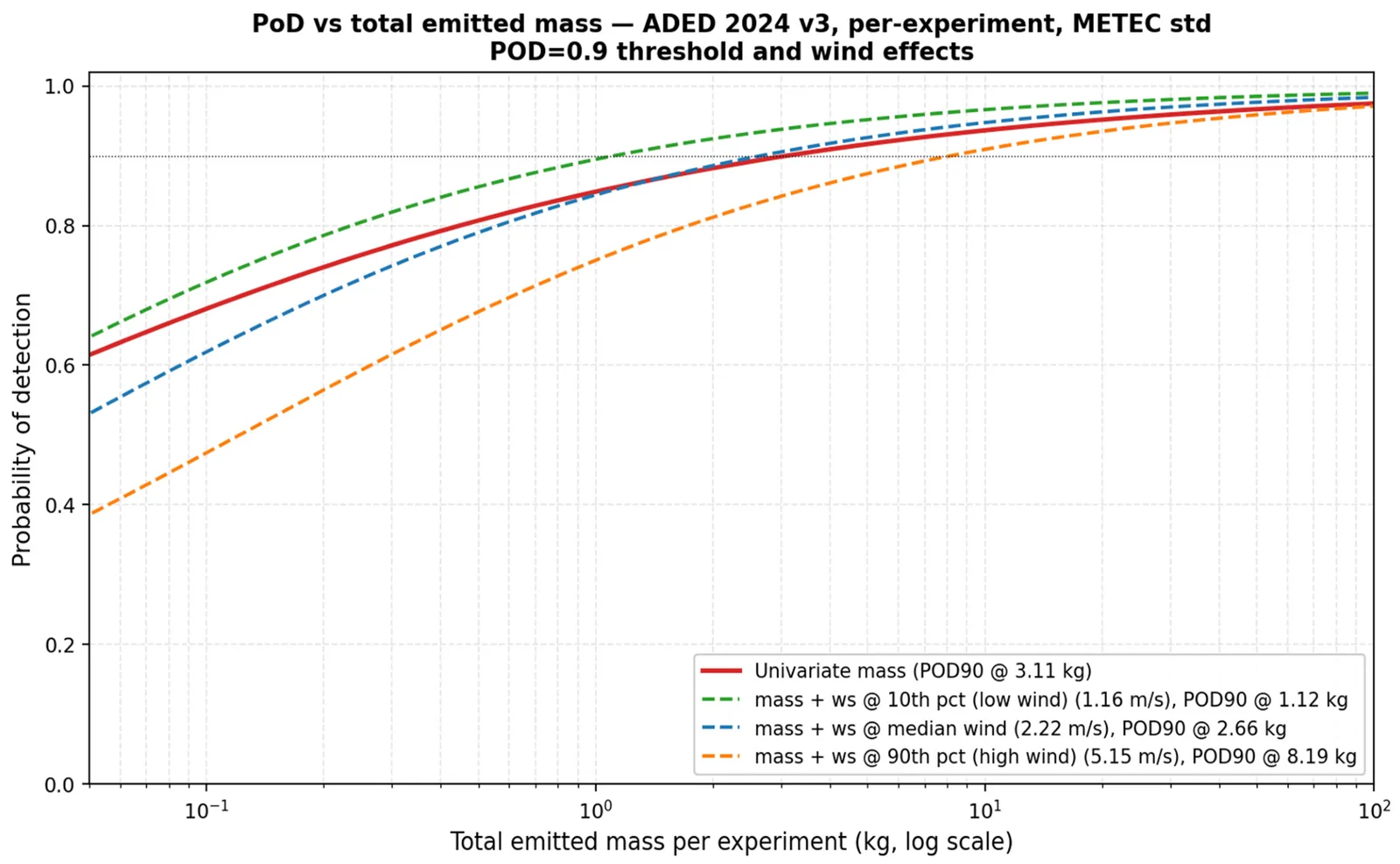
BluBird PoD as a function of the total mass of methane emitted per experiment. Curves show the univariate logistic fit versus total mass, along with multivariate fits of mass with wind speed.

Another way of approaching the combined effects is to consider how detection might depend on the total methane mass emission per experiment. This combines the influence of rate and duration and can help define detection rate in terms of a potentially useful additional parameter. For the ADED 2024 per-experiment data, the univariate relationship is significant but relatively weak (Figure 17). The 90% PoD is reached at an emission mass of around 3 kg of methane. When analyzed as a bivariate set of emission mass and wind speed, the estimated 90% PoD varies from 1.1 kg at wind speeds near 1.2 m/s (10th. percentile) to 8.2 kg at 5.2 m/s (90th. percentile). Overall, the total methane mass emitted per release is the strongest single predictor of detection. Release duration is the next strongest predictor, with release rate being considerably less significant. The relationships are weak, however, which, as noted earlier, reflects the fact that, for the BluBird CEMS, there is little variation in the detection rate throughout the range of conditions covered by ADED 2024.

### 3.4. Minimum Detection Level (MDL)

Here, we define the minimum detection level (MDL) for emission rate as the lowest reliably detected rate, encompassing the set of potential error contributors in the BluBird system. Based on the per-experiment trivariate PoD curves applied to release rate, release duration, and wind speed for the ADED 2024 data, and using the standard METEC 20-min start-time classification, the estimated MDL for typical ADED testing-day conditions is approximately 640 g/h, with a bootstrap 95% confidence interval of 1–1660 g/h. The MDL estimate improves if the additional potential detections under the 30-min start-time threshold or 75% overlap classification criteria are included (see Section 3.10).

### 3.5. Detection of Multiple Releases

The BluBird 2.0 real-time signal processing software available during ADED 2024 was not optimized to detect and quantify multiple simultaneous leaks (the 2.5 version currently used has been upgraded in this regard). However, as noted in Section 2.1, it was capable of generating more than one detection per experiment. These were then checked manually in a similar manner to Earthview’s current practice for assessing complex emission events reported for clients’ sites. With this approach, the BluBird system was correct 73% of the time in determining whether an experiment consisted of multiple releases. In other words, if the BluBird CEMS reported multiple detections, the experiment was indeed likely to be a multi-release event. For multi-release experiments, BluBird correctly identified the exact number of releases in 53% of the cases consisting of two releases. The success rate declined with an increasing number of releases (35%, 20% and 7% for 3, 4 and 5 releases, respectively).

### 3.6. Detection and Response Times

Temporal parameters of interest include the elapsed time until detection reports were issued (referred to as the Time to Detection in [20]), and how well the BluBird system detected the actual start times of releases. The former describes the time required to alert a client to the presence of an emission, while the latter is important for quantifying the total emissions from an event. For the ADED program, participants submitted initial detection reports and were then allowed an additional seven days to update these initial reports. For ADED 2024, the BluBird detection reports that were not subsequently updated (48% of the total number of reports) had a mean time to detection (i.e., the difference between the release start time and the time when METEC received the detection email from Earthview) of 31 min. (This is equivalent to how fast a BluBird client would be expected to receive an automated emissions-event alert). If an event required additional analysis by a staff member, then the median time to alert was about 49 h. This manual check was performed for 52% of the experiments. Typically, these were events for which the automated source location tools had difficulty in assigning sources with high confidence. All the reports were added together in [19,20], which yielded a mean of time to detection of about 31 h. In normal operation, in addition to the emission event alerts that are issued within about 30 min and which are based on emission rates, the BluBird CEMS can alert clients to high methane concentrations within about 1 min of the data acquisition.

The detection reports emailed to METEC included an estimated start time for the individual releases. The time difference between these BluBird-assigned start times and the releases’ actual start times for ADED 2024 had a mean offset of +9.3 min (BluBird earlier than METEC), with a median difference of −9 s. Seventy-eight percent of the BluBird-reported release start times were within ±10 min of the actual times, with 92% within ±30 min. Reported release end times in 2024 were similarly accurate; 73% were within ±10 min of the actual end times, with 83% within ±30 min. The manually determined start times for ADED 2022 were substantially less accurate; 46% were within ±10 min, with 66% within ±30 min.

### 3.7. Emissions Quantification

Quantification performance is assessed for the ADED 2024 program in terms of emission rates and total emissions averaged over different time intervals. The per-experiment and single-release subsets as classified using the 20-min and 30-min start time criteria are discussed here (results for 75% overlap detection classification criterion differ only slightly).

#### 3.7.1. Emission Rate

**Figure 18:**
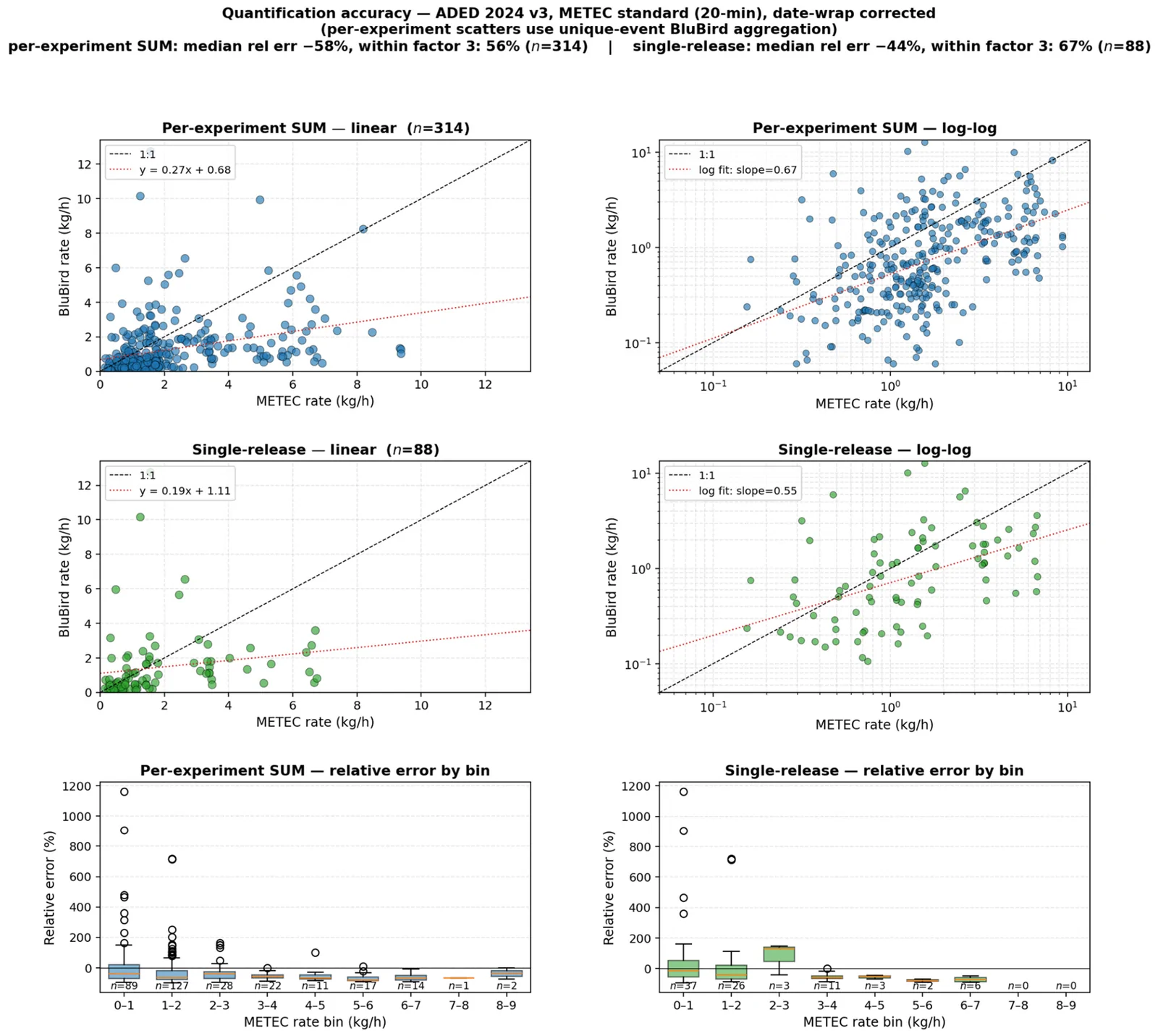
Comparisons of METEC ADED release rates (kg/h) versus corresponding BluBird-reported rates. The circle colors refer to per-experiment and single-release results.

Emission rates (Figure 18) show considerable scatter, as is typical of dispersion-model-based CEMS to date (e.g., [19,23]). Median relative quantification error as calculated for the per-experiment subset is −59% (*n* = 314; Figure 18). For single-release experiments, median relative error is −44% (*n* = 88). Error varies with release rate (Table 9), with 54% of estimated rates within 3x of the actual rates for the per-experiment subset and 67% for single-release experiments (Table 10). Results are similar for the other classification criteria subsets that include potential detections (the 30-min start time and 75% overlap criteria). For the per-experiment comparison using METEC standard grading, BluBird’s median relative error is −29%, with 56% of the rates within a factor of three.

#### 3.7.2. Total Emissions over Time

**Figure 19:**
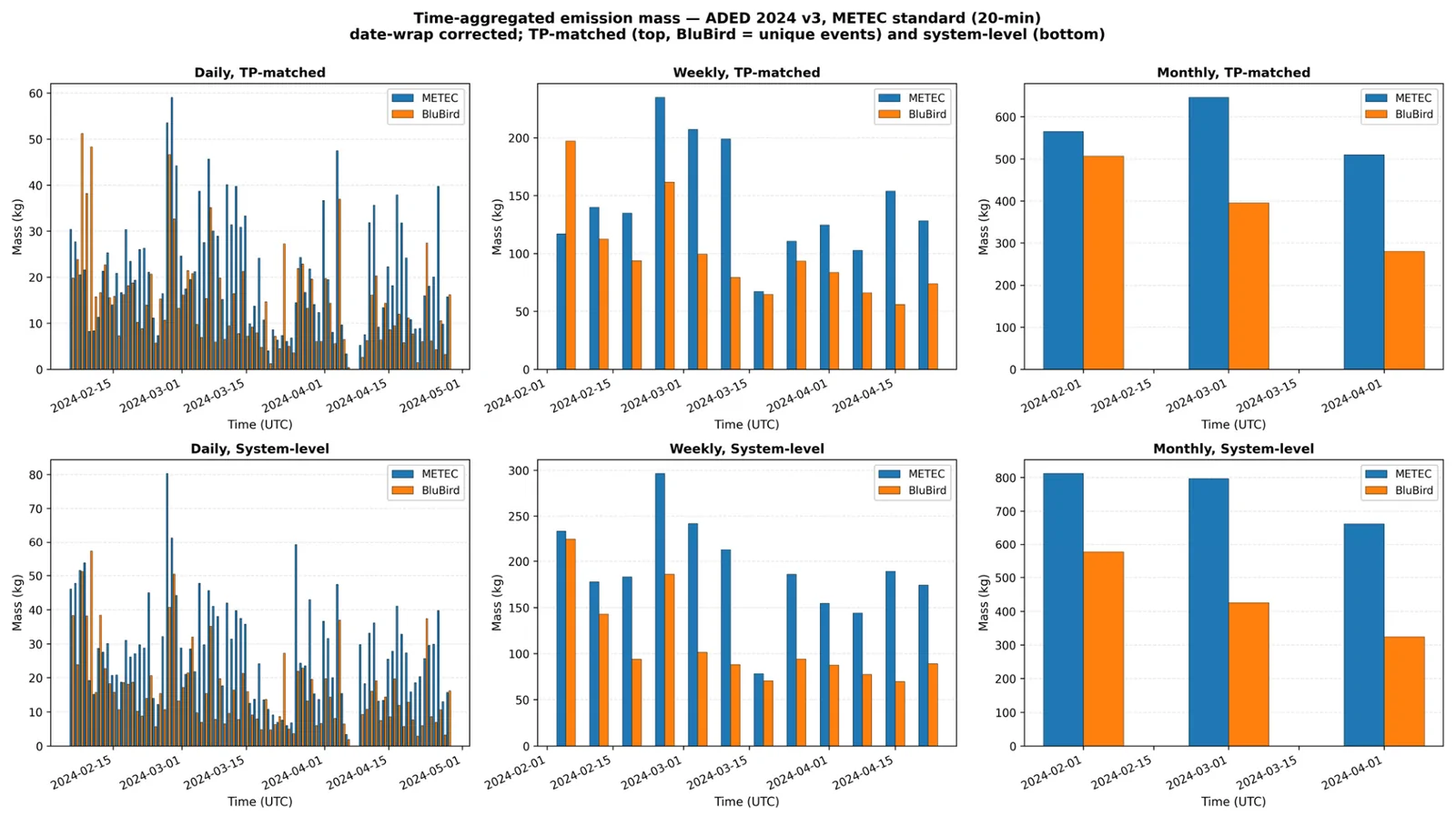
Comparisons of METEC-reported (standard METEC classification) and BluBird-estimated methane-emission total mass when summed over daily, weekly, and monthly intervals for the all-releases data set. The (**top**) panels are for successfully detected (“TP-matched”) releases. The (**bottom**) panels include every release (i.e., encompassing all TPs and FNs).

**Figure 20:**
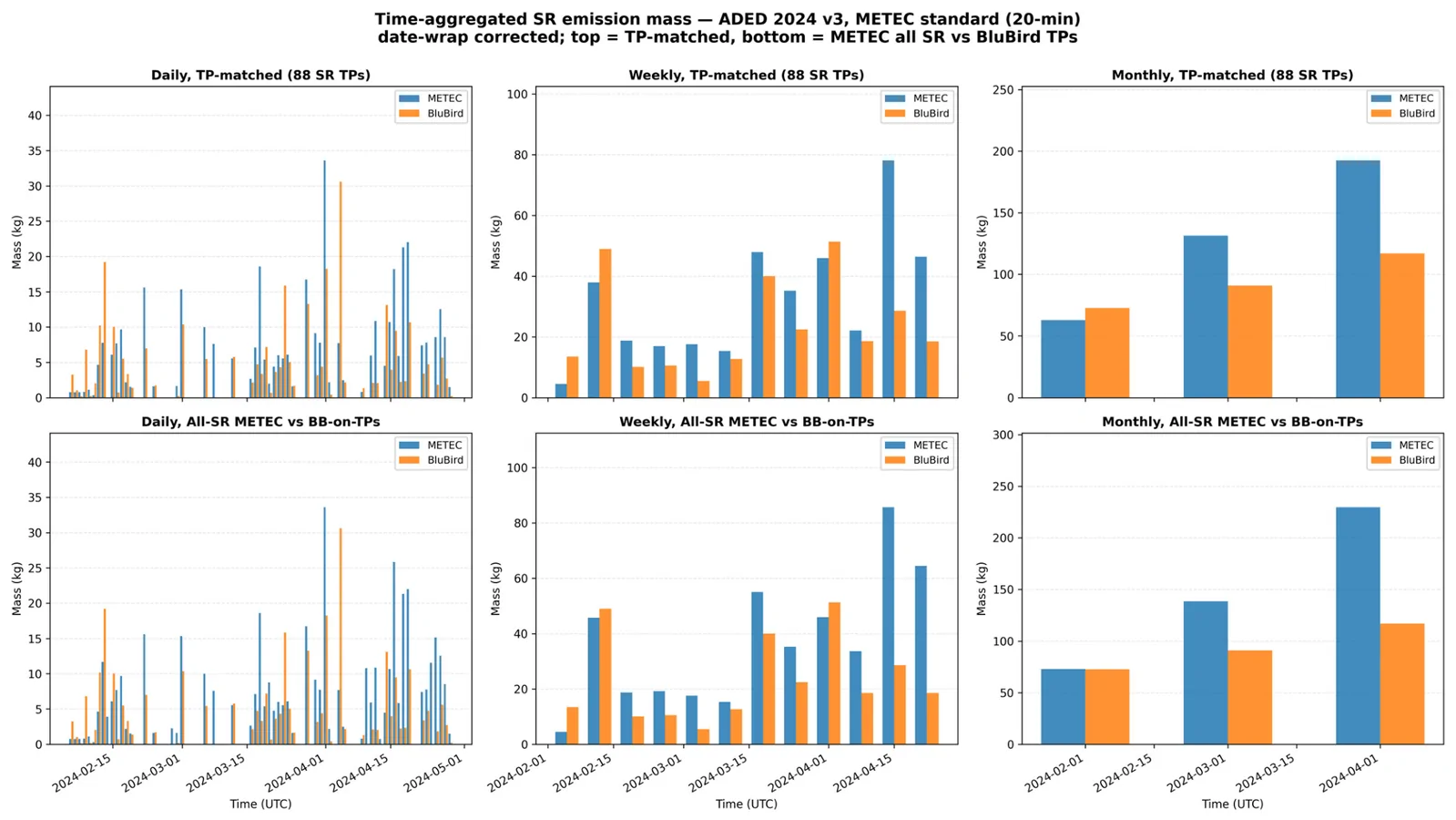
Comparisons of METEC-reported (standard METEC classification) and BluBird-estimated methane-emission total mass when summed over daily, weekly, and monthly intervals for the single-release subset of experiments. The (**top**) panels are for TP-matched releases. The (**bottom**) panels are for single-release experiments only (i.e., encompassing all TPs and FNs).

Estimated and actual methane emissions summed over daily, weekly and monthly periods during the ADED 2024 program are presented in Figure 19 and Figure 20. The “TP-matched” plots compare total emissions for releases that were detected by BluBird, and address the question of, “When BluBird detected releases, how well did it quantify the amount of methane released?” The “system-level” plots pertain to how well BluBird matched the total methane released, including from releases that BluBird did not detect. As expected, differences are larger when some releases during multiple-release experiments were not detected, or when releases were over-reported (the “system-level” panels in Figure 19). The similarity of the “TP-matched” and “system-level” totals for single-release experiments only (Figure 20) further highlights the impact of multiple releases on the comparisons. Overall, BluBird generally underestimated the total mass emitted.

**Figure 21:**
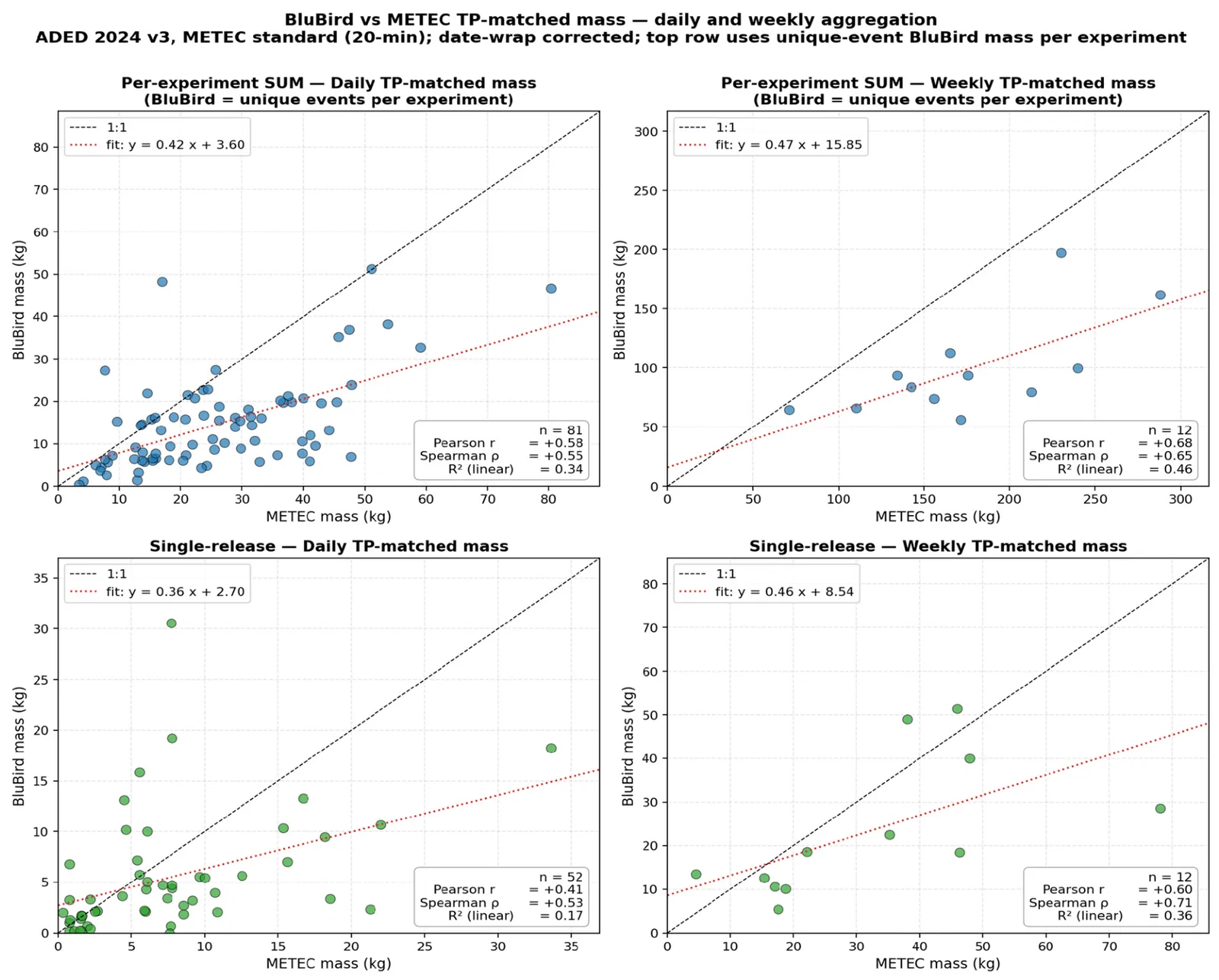
Relationships between METEC-reported (standard METEC classification) and BluBird-estimated methane-emission mass for per-experiment and single-release subsets when summed over daily and weekly intervals. The circle colors refer to per-experiment and single-release results.

The correlation increases when the TP-matched releases are summed over longer time intervals (Figure 21). For daily intervals, correlations are statistically significant for both the per-experiment (R^2^ = 0.34, *p* < 0.001, *n* = 81) and single-release subsets (R^2^ = 0.17, *p* = 0.002, *n* = 52). When summed over weekly intervals, per-experiment R^2^ rises to 0.46 (*p* = 0.02, *n* = 12) and single-release R^2^ rises to 0.36 (*p* = 0.04, *n* = 12).

Over the entire ADED 2024 program, METEC released a total methane mass of 2272 kg (a summation of all releases during the program). Using the METEC standard grading of the all-release data set, the total mass reported by BluBird was 1326 kg, or 58% of the total. About half of the difference is due to missed releases during the multiple-release experiments, with the other half being attributable to underestimation of emission rates. If the comparison is limited to just single-release experiments to avoid complications from the multiple-release detection issues, BluBird detected 64% of the total mass emitted (441 kg) for the standard METEC grading, or 74% using the 30-min classification criterion. With the single-release data set, we can address the question of how much of the underestimate of emitted methane is due to underestimating release rates versus failing to detect some releases. Of the methane mass that was not reported by BluBird for single-release experiments, only 3.8 kg (3%) is due to missed releases. The remainder (110.6 or 97%) is attributable to underestimating the actual emission rates for the 99 detected releases.

### 3.8. Identification of Emission Source Location

During ADED 2022 and 2024, natural gas was released from any of five equipment groups on the METEC site (Figure 2 in Section 2.1). For the ADED 2024 standard 20-min classification single-release subset, the BluBird CEMS correctly identified the emitting equipment group for 75.0% (66 of 88) of the experiments. Identification accuracy showed no apparent dependence on position on the site, or on wind conditions. BluBird was not able to reliably identify the specific piece of equipment (“equipment units”) within equipment groups; equipment units were correctly identified 18.2% of the time. This was expected since the spatial resolution of the grid system that BluBird used to identify potential leak locations was sufficient to resolve the separate equipment groups but not the individual equipment units within groups.

### 3.9. Influencing Factors

For the ADED 2024 single-release detection subset (*n* = 88), wind speed and release-rate quantification accuracy were significantly correlated, with accuracy increasing with increasing wind speed. Median error (BluBird minus METEC) decreases from −57% at winds < 1.5 m/s to about −45% for speeds between 1.5 and 3.5 m/s, with error dropping to −8% at intermediate speeds of 4.5 to 6 m/s. For winds stronger than 6.0 m/s, the error increases to −47%. Using the all-release data set, BluBird underestimated release rate across the wind speed range, but the error was greatest for releases at wind speeds below 2 m/s. When using the all-release data set, there were no clear patterns with wind speed, probably due to the confusing influence of multiple releases during the individual experiments. For low wind speeds, BluBird 2.0 applies an alternative dispersion model in place of the Gaussian plume model, but there are not enough of these cases to draw conclusions regarding the contribution of the low-wind model.

As noted earlier, one factor that may have affected detection rate indirectly through the METEC standard TP, FN, FP classification process is the potential for methane to have lingered on the METEC site in between separate experiments. Although wind speed alone did not correlate particularly strongly with detection rate, the combination of low wind speeds with short time gaps between experiments may have been a factor. For situations where the inter-experiment gap was less than 60 min and the mean wind speed was less than two m/s (a total of nine cases), BluBird assigned start times that were 100 min earlier than the METEC release start time on average. Sixty-seven percent of those cases were more than 60 min early. Outside of cases with short-gap plus low-wind conditions (106 cases), the median BluBird-reported start time was only 12 min early, consistent with the 10-min time stepping used by BluBird’s automated detection software (Section 2.1).

**Figure 22:**
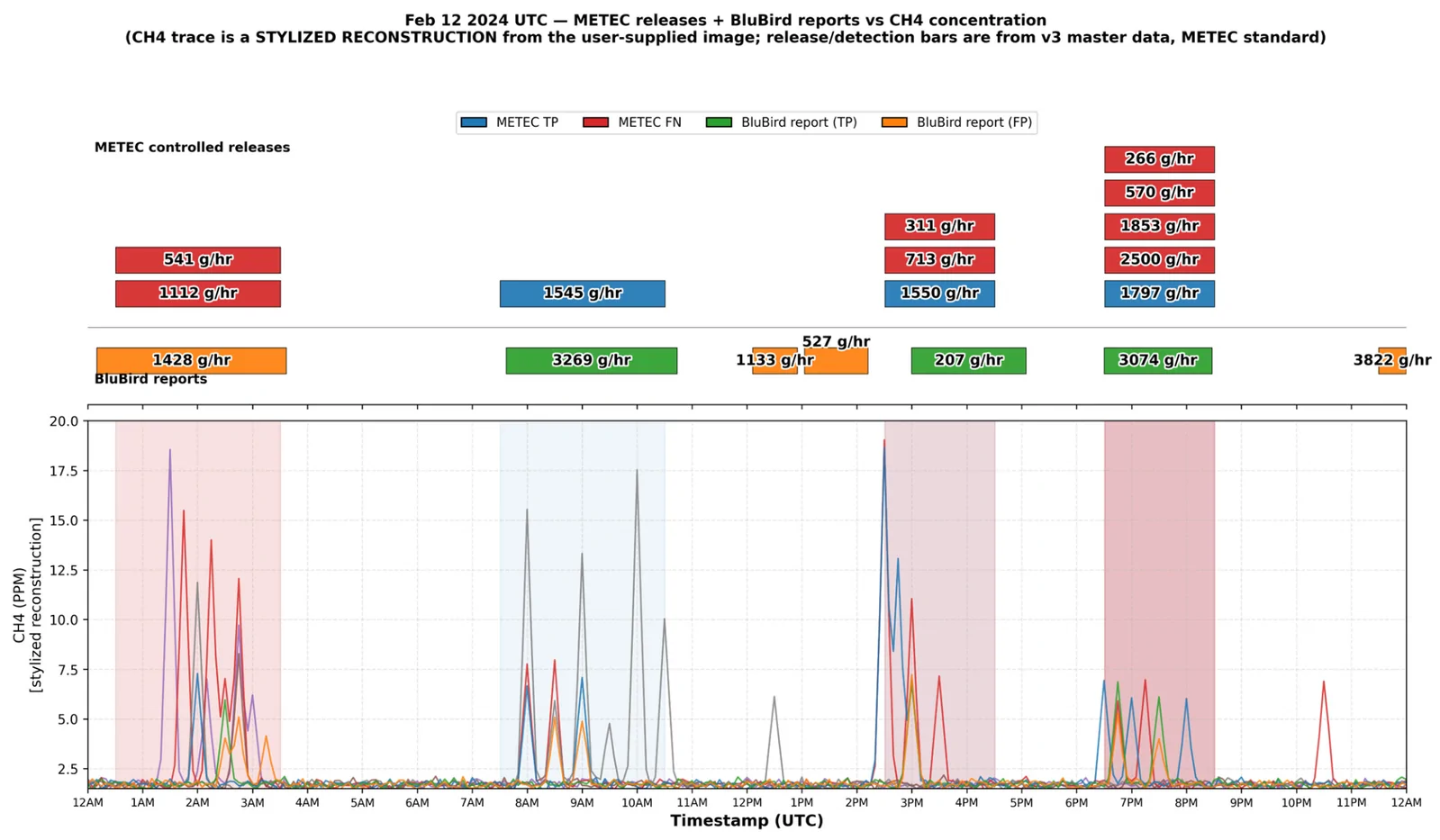
Correspondence of METEC releases and BluBird detection reports (**top panel**) and estimated methane concentrations (**bottom panel**) taken from the Earthview data dashboard for 12 February 2024. Note the elevated concentrations from 12 pm to 2 pm and from 10 pm to 11 pm, which did not correspond to METEC releases. In the top graph, the blue and red colors indicate whether the release was considered detected (blue) or missed and classified as a false negative (red). The green and orange colors indicate whether the BluBird report was graded as a detection and classified as a true positive (green) or as a report that had no corresponding METEC release (orange). Multiple bars in a stack indicate multiple releases during a single experiment. Each of the lines in the line graph represents concentrations estimated by for an individual BluBird node, with each node’s data shown in a different color. The shaded areas correspond to start and end times for ADED releases.

This suggests the possibility that at least some of the BluBird detections that were rejected under METEC’s standard 20-min start time threshold, as well as some false positive reports, may have been influenced by residual methane, either by (a) BluBird seeing above-background methane concentrations just before a new METEC release and then treating that as part of the subsequent release, (b) not seeing a clear-cut return to background conditions during short gaps between experiments, or (c) treating the elevated concentrations as releases on their own, resulting in FPs. There are several examples of these situations apparent in the BluBird-reported methane concentrations. For example, Figure 22 shows the METEC releases and BluBird reports for 24 h starting on 12 February 2024 00:00 UTC, superimposed on the time series of BluBird-estimated methane concentrations (this event was introduced earlier in Section 3.1). The first BluBird report’s start time of 00:10 was 20 min and 37 s earlier than the corresponding two METEC releases, resulting in one FP and two FNs. At 00:10, two of the BluBird nodes reported concentrations of 2.4 ppm, compared to usual background values around 1.9 ppm (for example, the inter-release period from 04:00 to 06:00). At 06:30, three nodes reported concentrations ranging from 2.3 to 3.2 ppm but the duration was too short to trigger a release detection. From 12:00 until 14:00, concentrations are well above background, resulting in BluBird issuing a detection report for a time when no METEC release was active. Finally, at around 10:20 pm, concentrations were well above background, with multiple nodes reporting between 5 and 6 ppm methane. Again, there is no corresponding METEC release around this time. Winds were near-calm on 12 February, with a neutral to stable boundary layer, conducive to gas trapping and a lack of clearing of residual gas. The data suggest that a time gap of ≥2 h between experiments, or wind speeds greater than approximately 2 m/s, is enough to clear the site of residual methane. Neither time gap or wind speed individually accounts for the relationships seen. This pattern of more variable concentrations during experiment gaps can be compared to the more typical situation shown earlier in Figure 7, where concentrations during the gaps are more similar to normal background conditions.

### 3.10. Sensitivity to Detection Classification Criteria

It is useful to consider to what degree the overall interpretation of CEMS performance is affected by relatively slight changes in the rules used to decide if a detection report classifies as a true detection. As discussed earlier, outside of the ADED grading protocol, it can be argued that the BluBird CEMS detected more releases than are included within the specific ADED-defined results. For example, the overall detection rate for ADED 2024 experiments (i.e., per-experiment detection) was 91% using the METEC standard 20-min start-time threshold, but this increases to 94% for the 30-min start-time threshold and 97% for the 75% overlap criteria due to the inclusion of more potential detections. Using these criteria, the number of FNs and FPs that do not overlap at all with METEC-reported releases is small; three out of the 775 ADED 2024 releases were totally missed (0.5%), in that BluBird reported no corresponding detections within one hour of the release, and just three detection reports were issued for times when no release was underway (defined as within ±60 min of a release).

Application of the 30-min start-time threshold re-classifies relatively few FNs as TPs. However, three of these TPs represent releases at rates greater than 6 kg/h, which lie at the far right of the release rate distribution. This small change in classification has notable effects on PoD curve fitting (Table 6 and Figure 14). For the “all releases” data set, the 90% PoD decreases from 16.45 kg/h (outside the ADED test-rate range) to 9.04 kg/h using the 30-min start-time threshold and to 3.94 kg/h using the 75% overlap threshold. For single-release experiments, the 90% PoD rates improve from 1.59 kg/h with the standard METEC grading to 0.26 for the 75% overlap criterion. As discussed earlier, though, switching from the all-releases data set to the per-experiment data set is the most significant factor, independent of the detection classification criteria used, with the 90% PoD level decreasing from 16.45 kg/h for all releases under the standard METEC grading to 0.64 kg/h for detection of individual experiments.

To assess the sensitivity of the PoD estimate to the curve-fitting methods and detection classifications, we consider two additional approaches. First, for its 2025 report [21], METEC used a Weibull-form exponential curve fit (PoD = 1 − exp(−a · x^b^) to detection as a function of release rate. Applying this method to the BluBird results for the ADED 2024 30-min classification of the all-releases data set yields a 90% PoD of 9.21 kg/h, essentially the same as the 9.03 kg/h value estimated using the trivariate logistic regression approach. Second, we can apply a different detection classification method. For this, we classify each BluBird detection report as a TP or FN based on whether it has a Jaccard Similarity Coefficient index ≥ 50% (see Section 3.1.2). When applied to the per-experiment data set, the 90% PoD is 0.27 kg/h, and 4.3 kg/h for the all-release data set. The 90% PoD value of 0.27 kg/h is within 8% of the 90% PoD value listed earlier when the 75% overlap classification criterion is used. Again, the choice of which basic assessment approach is being used (i.e., whether to grade based on detecting all releases or detecting individual experiments) outweighs the specific choices concerning curve fitting or detection classification.

**Figure 23:**
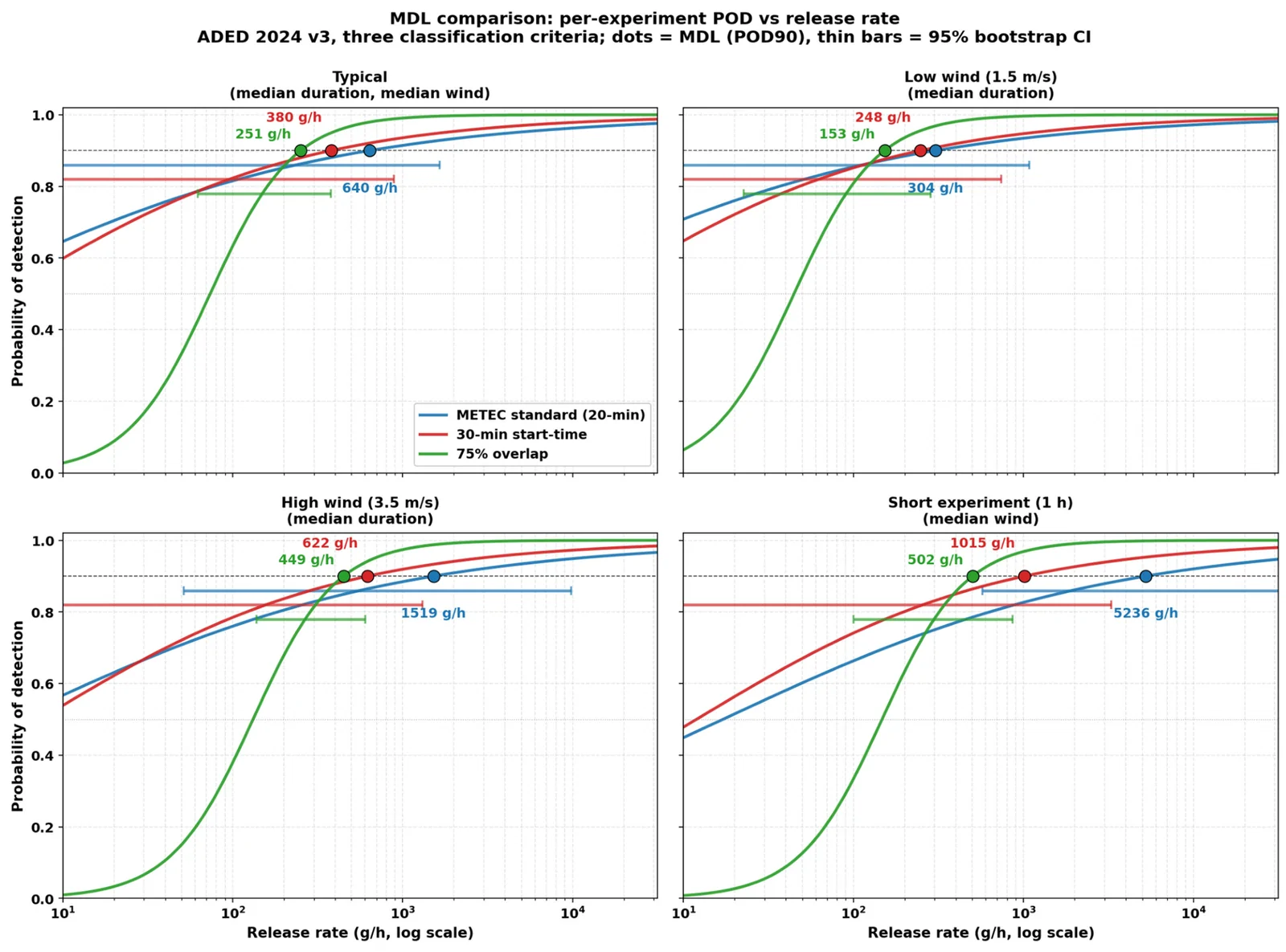
Minimum detection levels and detection-level ranges under different test conditions, for detections classified using the METEC standard 20-min start-time criterion and for potential detections using the 30-min start-time and 75% overlap criteria.

Figure 23 shows the estimated MDL levels for the different detection classification criteria. With the 75% overlap classification, that estimated METEC standard-classification MDL of 640 g/h decreases to approximately 250 g/h, with a 95% confidence interval of 50 to 380 g/h, for typical ADED testing-day conditions. More generally, across a range of wind speeds (1.5–3.5 m/s) and experiment durations (≥1 h), the MDL is roughly 150–500 g/h for the ADED 2024 test scenario. Using the 30-min start time threshold, the MDL is slightly greater at 380 g/h.

For the other measures considered—quantification, localization, and detection timing—there is little or no change resulting from using the alternative detection classification criteria.

## 4. Discussion

Using METEC’s standard grading of ADED program results, 85% of the false negatives (FNs) assigned to the BluBird CEMS for ADED 2024 were due to under-reporting the number of releases during multiple-release events (“experiments”), while 94% of the false positives (FPs) were excess reports of releases during an experiment. Accordingly, BluBird’s overall detection percentage, and emission rate corresponding to a 90% Probability of Detection (PoD), differ substantially if “all-releases” and “per-experiment” detections are considered separately. When the BluBird CEMS missed a release, it was, in nearly all cases, one of several during a multiple-release experiment rather than a completely missed experiment. And when a detection was reported that did not match an actual release, it was nearly always an over-reporting of releases during an experiment. In practical terms, if the ADED program had been an actual oil and gas production site, there would have been relatively few cases where the CEMS failed to alert a client that there was at least one leak on their site, and relatively few cases where an alert would have been issued when no leak was actually present.

If we focus on the “detection of all releases” in the ADED results, which is the approach used in METEC’s publications, then we can directly compare BluBird’s performance with other ADED participants. Of the 13 technology participants in ADED 2024, summarized in [19], of which 10 are point sensor networks (PSNs), BluBird (“Participant C”) ranked third among all participants for percent of true positive detections (TPs), with all but one of the PSNs scoring considerably higher than the scanning/imaging solutions. The BluBird CEMS also ranked third for mean quantification error over a range of 0.1 to 1.0 kg/h release rates (1st. for median error); third for quantification error for rates > 1 kg/h (5th. for median error); fourth for time to detection; sixth among the 10 point-sensor networks for false positive percentage; and seventh among the point sensor networks for source localization (with the scanning/imaging solutions outperforming most of the PSNs). Using these all-releases results, the BluBird PoD did not reach 90% within the range of releases tested (six of the 10 PSNs did not reach 90%). This compares to a 90% PoD of 0.64 kg/h for BluBird when the per-experiment results are used (see Section 3.3).

This very large difference in PoD figures highlights the main point of this study, which is that the way in which ADED results are analyzed and reported is significant—the PoD results presented by [19,20,22] and elsewhere to date present only a partial picture of detection performance. It would be valuable to see if applying this same distinction of “all-release” versus “per-experiment” to the other ADED participants’ detections would yield a similarly different view of detection rate and estimated 90% PoD. This is important since, as noted earlier, METEC’s ADED reports carry considerable weight for providers and users of methane monitoring systems, as well as for regulatory agencies and gas certification organizations.

The graphical overlay analysis used here suggests that a substantial number of BluBird release detections that were scored as false negatives (FNs) or false positives (FPs) under the standard ADED protocol actually correspond well with ADED releases in terms of start and end times and percent overlap. The correspondence is sufficient to reasonably conclude that BluBird detected the specific releases. Aspects of these detection reports (such as earlier estimated release start times) did not meet METEC’s ADED 2022 and 2024 protocol criteria, however, and thus cannot be said to have been misclassified (Section 2.4.2). The number of these rejected reports is sufficient to significantly affect the release detection ratio and detection pairings, especially for experiments with multiple releases where the start-time offset might result in multiple FNs and FPs for a single experiment. For example, the detection rate increases from 91% to 94% if slightly earlier reported release start times (the 30-min start time criterion) are allowed, and to 97% if a report is allowed if it substantially overlaps an actual release (the 75% overlap criterion). In terms of the ADED assessment criteria, this is simply a change in classification methods rather than an improvement in how the system itself performed. However, the improvement is still relevant when considered in broader terms outside of the ADED program itself.

It is important to point out that this sensitivity of technology performance assessments to ADED classification protocols is being addressed as part of the revised ADED 2025 guidelines. In METEC’s updated protocol, ADED participants will be able to specify detection criteria that are consistent with their particular technologies [24]. This should address the distinction between “per-release” and “per-experiment” detection, as well as allowing for quirks in processing such as the start-time offset inherent in the BluBird 2.0 system.

For the data considered here, the estimation of the 90% PoD level for gas release rates is quite sensitive to relatively small changes in the distribution of detections across rates. For example, relatively few additional detection reports were reassigned as TPs (13 in total) if the standard ADED start-time threshold for acceptable reports was increased from 20 min to 30 min. However, several of these additional TPs are for high-rate releases that lie at the extreme end of the PoD distribution curve. This has little effect on the 90% PoD estimate for the per-experiment data set. However, these additional detections have a large and statistically significant effect on curve-fitting to the all-release set. Compared to an estimated 90% PoD (16.4 kg/h; which lies outside of the ADED testing range) for the 20-min start time threshold, the all-release 90% PoD decreases to 9.04 kg/h if the extra 10 min of start-time offset is allowed, and to 3.94 kg/h when additional detections are allowed based on overlap of reports with release periods.

It is useful to consider how detection percentage aligns with emission release rates listed in active or proposed methane-mitigation regulations. For emission rate bins defined as ≥0.4 kg/h (U.S. EPA OOOOb) and ≥0.1 kg/h and ≥5 kg/h (U.S. EPA alternative monitoring methods for periodic screening technologies), detection rates for the three different detection criteria and the per-experiment data set are above 90% for each of the three EPA rate categories, except for the standard METEC-graded subset (20-min start-time threshold) at the ≥5 kg/h bin, for which the detection rate is 88%. However, including the few additional high-rate releases noted above that had slightly early assigned start times raises the >5 kg/h detection rate to 95% and 100% for the 30-min and 75% overlap classification criteria sets. Again, this highlights the significance of relatively slight changes in how detections are graded.

For ADED 2024, release duration had the strongest correlation with detection rate, while release rate showed the weakest influence overall, although correlation increased at higher wind speeds. Among other factors that are likely to affect detection, total emission per release (a combination of rate and duration) is a stronger predictor than rate alone for the ADED 2024 suite of conditions. The main factor driving detection is likely to have been the length of time required for an emissions plume to intersect a sensor, which is a function of release duration, wind patterns, and sensor positioning and density [50,51,52].

In terms of other influencing factors, no statistically significant relationships were found between boundary layer conditions and detection rates or emission quantification errors. Atmospheric stability as estimated using HRRR model fields did, however, correlate with some of the cases where BluBird reported earlier release start times or failed to detect gaps between METEC releases, which we attribute potentially to methane gas lingering on site in between experiments (see Section 3.8). This “residual methane” possibility is only a theory; we have no access to independent data to confirm or refute it. It would be a valuable exercise for METEC or other ADED participants to see if similar patterns of slightly elevated concentrations between experiments are present in their observations. In any event, in terms of how well the system performed under ADED rules and conditions, these cases remain as misclassifications since it can be argued that even if residual gas is present, it represents an ADED background condition for which a participating CEMS might be expected to account.

Any information a CEMS can provide about leak source location is potentially useful for LDAR efforts. This is also true for leak start and end times, which can help operators target particular equipment types or assess effectiveness of repairs. For ADED 2024, BluBird correctly identified the emitting equipment group in most cases, and 92% of the BluBird-reported release start times were accurate to within 30 min. It is worth noting that, although BluBird 2.0 as deployed during ADED 2024 was not optimized for multiple-release detection, it proved to be able to identify, in 73% of cases, when an experiment consisted of more than one release. Given this, emission detection alerts could be modified to inform an LDAR team to look for more than one leak, even if the exact number and location of those leaks is not known.

BluBird’s median release-rate quantification error for the ADED 2024 per-experiment subset is within approximately ±50%, with 72% of estimates within 3x of the actual rates, with a negative bias in rates across the full range of release rates tested. Methane mass emitted over time is, ultimately, the main parameter that methane monitoring systems are intended to measure and thereby influence. Converting the BluBird ADED measurements into totals of emitted methane summed over different time periods yields comparisons that are relatively direct and straightforward to interpret. Totals summed over daily and weekly periods are significantly correlated, which suggests that such totals can be used to monitor trends. Averaging emission mass estimates over time improves accuracy relative to METEC release mass.

These methane mass comparisons highlight BluBird’s tendency to underestimate the ADED emissions. Some of this underestimate in total emissions arises from the fact that fence-line sensors do not detect 100% of emissions from a site [25,53,54]. However, since the large majority of BluBird’s underestimate is due to missed detections of releases during multiple-release experiments, this spatial sampling issue appears to be relatively minor. Only 3% of the underestimate in methane-emission mass for the single-release experiment subset is due to missed detections; this suggests that the underestimate in emission mass is due to rate bias rather than to missed releases. Given this, work on improving the BluBird system should prioritize emission rate quantification rather than leak detection capability.

Based on our experience with field deployments and testing, the main strengths of the BluBird CEMS are moderate cost, durability in the field, consistency of performance, ease of deployment, and effective emission-detection capability. Limitations include gaps in cellular coverage, battery fatigue due to numerous charging cycles, water ingress through the air intake, and vulnerability to damage from animals. These continue to be addressed over time as new iterations of the BluBird hardware are deployed. The most significant limitation is the accuracy of emission rate quantification. Fortunately, several promising routes exist for improvement. The Gaussian plume modeling we presently use is computationally efficient, which is a requirement when calculating emission rates for hundreds of sensor nodes at 30-s intervals. However, it embodies some important physical assumptions, such as sufficient travel distance for plume mixing, applicability during low and high wind speeds, and suitability of empirically derived dispersion coefficients. These assumptions are routinely stretched, if not violated, in our field settings. We are working to address this by augmenting or replacing the basic Gaussian plume approach with a Gaussian puff model [55], which will provide more options for predicting plume behavior.

Quantification error is driven by sensor and algorithm performance and by factors associated with the conversion from concentration to emission rates. In addition to upgrading the plume model, improving source localization is perhaps the most direct step, since distance of plume travel becomes more accurate. However, in the ADED data, we found no significant relationship between quantification accuracy and the distance between source and sensor. Detection of multiple sources should also contribute quantification accuracy; as seen earlier, accuracy was improved for single-release versus multiple-release experiments. Presumably, the accuracy statistics could also be improved if stricter criteria were applied to exclude observations that are particularly challenging for the dispersion modeling. Presently, all observations are quantified, but from the results presented earlier in Section 3.9, excluding observations during near-calm conditions and the highest wind speeds should be expected to reduce mean error. For the ADED 2024 results, though, excluding winds below 1.5 m/s or above 6 m/s had a minimal impact on median error. As noted earlier, no significant relationships were seen between quantification accuracy and other factors such as estimated surface-layer stability or variability in wind direction (a proxy for plume meander). The fact though that the emission rate error shows consistent biases for different wind speed ranges suggests that simple adjustments to the plume model’s prescribed dispersion parameters based on wind speed would reduce the error range. Increasing dispersion at wind speeds of less than 2.5 m/s and decreasing dispersion at wind speeds greater than 4 m/s would increase the estimated emission rates at the lower speeds and decrease them at the higher speeds, bringing the estimated emissions closer in line with the actual emission rates during ADED.

In terms of quantifying total emission mass, deploying more sensors on site is an obvious step, but we are investigating additional options such as dividing sites into virtual sub-grids, which constrains source location solutions to smaller areas of a pad. Learning approaches (e.g., [56,57]) that can extract patterns from data time series are also fruitful lines of research. These tools may provide a potential alternative to the classical machine learning methods used to create the digital twin models in the BluBird system, or to replace the statistically derived response functions between MOS resistance and methane concentration. One constraint on the use of deep learning for these aspects is the availability of training data sets that encompass a broad enough range of conditions and that account for combinations of methane concentration, non-target gases, and atmospheric conditions, along with slight but significant differences between individual production batches of MOS sensors.

**Figure 24:**
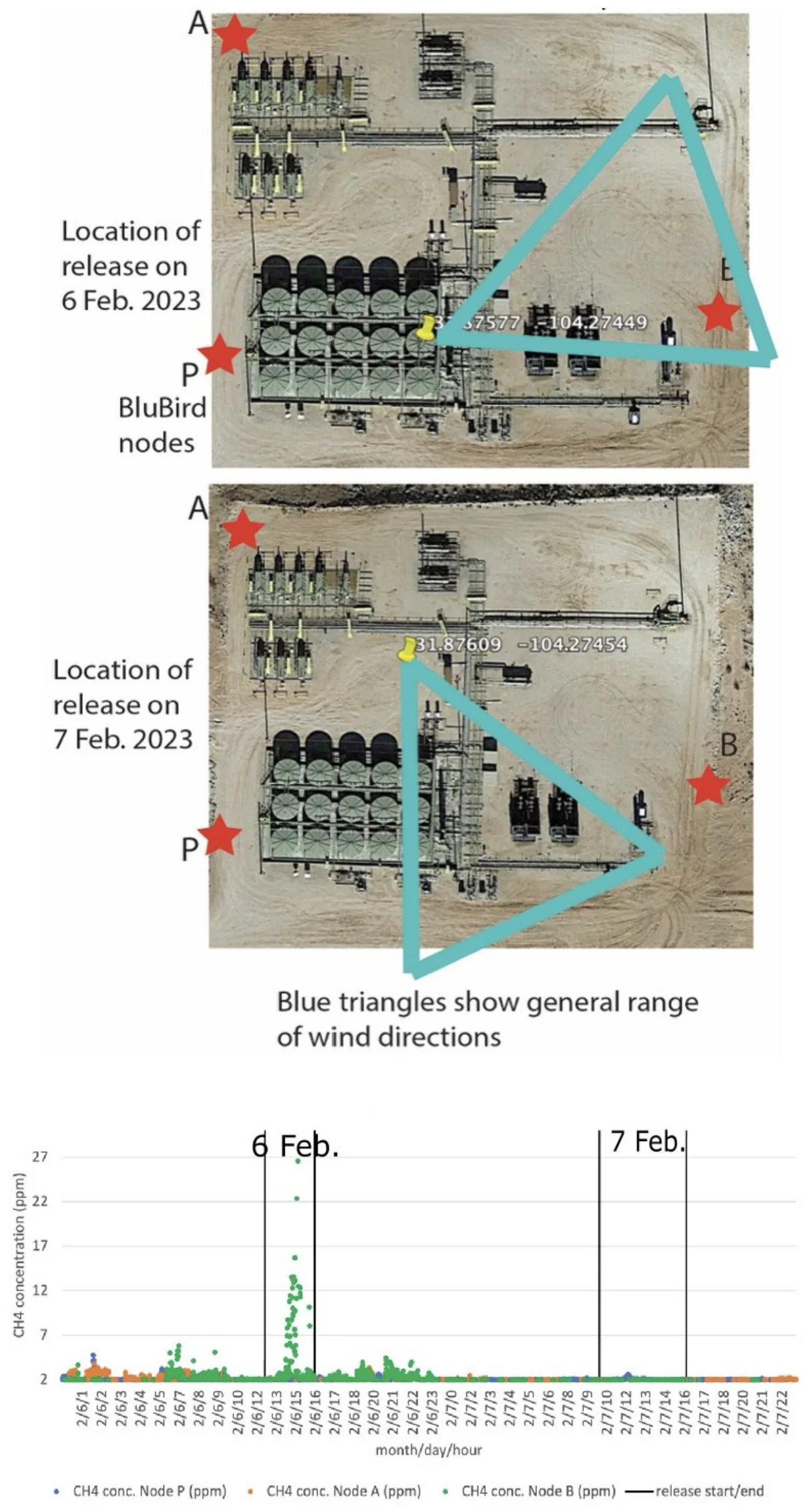
BluBird deployment and concentration measurements at one of the controlled-release sites described in [15]. The (**top**) panel indicates locations of BluBird sensors (red stars) on 6–7 February 2023. The yellow pins are the source locations of METEC’s gas releases, while the blue triangles indicate likely plume directions during the releases as estimated based on wind directions (i.e., winds were from the southwest on 6 February 2023 and from the north-northwest on 7 February 2023). Methane concentrations reported by the three BluBird sensors are shown in the (**bottom**) panel, with the approximate release durations bracketed by the black vertical lines. Wind speeds during the test releases averaged 5 to 6 m/s during the release period, with relatively constant wind directions of about 225 degrees on 6 February and 320 degrees on 7 February. Methane concentrations spiked on 6 February at Sensor B, which is downwind from the release point. A small spike of about 0.6 ppm is reported by Sensor P on 7 February during the release period. The relatively high wind speeds would typically result in a narrow, jet-like plume, which would have reduced the likelihood of the plume intersecting one of the few sensors on site.

More work is needed to understand how well these and other programs’ test results capture the performance of CEMS in real-world situations, and why results may differ from one test program to another. For example, during a METEC-conducted field test consisting of controlled gas releases at operating oil and gas pads [15], in contrast to their ADED performance, none of the participating technologies yielded a PoD reaching 90%. However, a key point made by [15] is that, when an emission source was upwind of a sensor, the sensors typically detected an increase in methane concentration. This is demonstrated in Figure 24, which shows results from Earthview’s participation in that field-test effort, where BluBird sensors were deployed at two of the 11 sites used.

In this case, as well as others during the tests, the BluBird sensors show enhanced methane readings during METEC’s test releases in each case when a sensor was downwind of the release, consistent with [15]. Here, the discrepancy between detection performance during ADED and in these field tests is a function of the issue of sensor distribution and density (i.e., three sensors were deployed in this case, compared to 12 during ADED 2024).

When comparing test results in this way, it is important to reiterate that ADED is carried out under controlled test-site conditions and is limited to the setting and weather conditions at the northern Colorado METEC location. While METEC has added realism to the test facility by having actual production equipment in place, real-world oil and gas sites can be larger and more complex in many cases. Some, such as compressor stations, have equipment and operating modes that are not yet represented during ADED, and the nature of leaks, such as variable rates and intermittency, may be more complicated. Other factors such as more varied terrain, vegetation, and surrounding structures will also differ from site to site and between regions. Caution is therefore needed when extrapolating the ADED performance results to particular locations and conditions. To complement ADED and similar programs, a formal single-blind testing regime that could extend the ADED protocol to operating sites, over sufficient time spans, for different environmental settings, and that is practical for the operators and affordable to the CEMS technology participants would be ideal. In the meantime, additional informal field testing of the BluBird CEMS consists of coordinating with operators to validate leak presence and emission rates in response to leak alerts at actual sites, along with maintaining a log of events such as hung dump valves that were detected by the CEMS and confirmed by the site operators.

If site emission totals and/or rapid detections of emissions are priorities, then the number and distribution of point sensors must minimize the time required for the sensor to observe an emission. This is the approach that most technologies probably took for their ADED participation, and in most cases at least, it can explain why their performance differs so much between ADED and the field tests summarized by [15]. To achieve a high likelihood of detection at a given emission rate, a CEMS taking part in an ADED program has to both minimize the time required to observe a leak and maximize the classification algorithm’s capability. On the other hand, for a periodic survey method, only the second part—the algorithm performance aspect—is typically quantified. For example, an aerial survey company might refer to a 90% PoD level of 2 kg/h, but that is the likelihood of detection assuming that the aircraft is overhead. A CEMS that achieves a 90% PoD of 2 kg/h as assessed from the ADED results can claim a 90% probability of detecting any leak at any time, based on the range of conditions encompassed by the ADED program. However, a periodic survey technology that offers a 90% PoD for 2 kg/h is typically making that claim only for the cases where the survey coincides with a leak. That such PoD values refer to specific survey missions (or, in the case of an on-site inspection, a visit from an optical gas imaging team) seems obvious and is typically clear in related publications (e.g., [58]), but for the general public, one type of PoD might seem the same as another.

Another way of describing this issue is to treat the probability of detecting an individual single leak as a function of the probability that the sensor and leak intersect (either physically or via remote sensing) multiplied by the probability that the sensor’s algorithm detects and alerts to the leak. Or,

*P*_(detect)_ = *P*_(intersect)_ × *P*_(algorithm)_

For a CEMS point sensor network, *P*_(intersect)_ will typically be roughly 30 to 90%, depending on sensor density and wind direction variability, with a *P*_(algorithm)_ of maybe 80%. For a periodic survey, *P*_(intersect)_ depends on the amount of time the survey is on site relative to the duration of a leak. As an example, for a single 2-h leak and a quarterly OGI inspection, *P*_(intersect)_ is about 0.1% (2 h/[~90 days × 24 h)]). Assuming a *P*_(algorithm)_ of perhaps 95% for the OGI inspection, the probability of detecting any single 2-h leak is therefore about 40% for the CEMS and about 0.1% for the OGI survey. This simply illustrates the point that it is important to recognize the type of detection requirement and what type of PoD is being discussed when comparing different testing programs and technologies.

## 5. Conclusions

Testing of continuous emission monitoring systems (CEMS) designed to detect, quantify, locate and report methane emissions under representative oil-and-gas-site field conditions is a significant challenge. Here, using CSU METEC “Advancing Development of Emissions Detection” (ADED) test results for 2022 and 2024, we assess the performance of the commercially available BluBird metal-oxide based point sensor system.

Overall, BluBird performance improved substantially from ADED 2022 to ADED 2024. Emission detection reports show consistent overlap with ADED 2024 release periods, and the CEMS was able to identify short-duration releases and short gaps between releases. The system detected 91% of ADED 2024 experiments (defined as the detection of at least one release during an experiment), increasing to 97% when reports with slightly early start times were accepted. Detection rates for all releases during an experiment are 69% and 74%, respectively. The system effectively located sources, with minimal delay in time to detection and reporting.

The emissions rate corresponding to a 90% probability of detecting at least one release during an experiment is estimated as 0.64 kg/h using standard METEC grading, while in terms of detection of all releases in an experiment (the approach used by METEC prior to 2025), the 90% PoD ranges from 3.94 kg/h to 9.04 kg/h. For the release rates seen during ADED 2024, the estimated PoD for the BluBird system is only marginally influenced by rate; instead, it is dominated by how many releases are in multiple-release experiments. When a release went undetected, it was typically one among several in a multiple-release experiment. These differences in detection rate and PoD, depending on whether the measure used is the ability to detect the presence of one or more leaks during an emissions event versus identifying all leaks that are present during an event, are substantial. Summarizing emissions in terms of total methane mass released suggests that aggregating over time reduces overall noise [54,59]. Estimates of mass are biased low but benefit from time averaging, and are correlated significantly for weekly totals and could be useful for tracking trends or shifts in emissions from a site. 

While the ADED program, particularly with the newly revised ADED 2.0 protocol, is well suited to meeting its goals (see Section 2.2), there are several ways in which ADED and METEC could further assist sensor developers and potential users of emission monitoring technologies. To date, METEC has assessed the performance of emission monitoring systems using the “all-releases” approach (e.g., [18,20]), which as noted above, does not differentiate between missing an active-site (“site-level”) leak or missing one leak among many simultaneous leaks. Point-sensor CEMS detect emissions on a continuous basis but are typically not optimized for identifying multiple, simultaneous leak sources. Imaging systems can see multiple sources but with less frequent sampling of the full site. Reporting results on a per-experiment basis in addition to an all-releases basis would place different technologies on a more uniform playing field, and would help in understanding their strengths, weaknesses, and best routes for improvement. Including occasional longer-duration releases at fixed rates from single sources would also make the ADED data more useful for testing and refining detection algorithms and plume modeling.

For the BluBird CEMS, the ADED data suggest that further improvements should focus on rate quantification [5]; other factors, such as leak detection and mapping of leak duration, contributed only slightly to overall error in these tests. Several routes exist for improving the estimation of emission rates as part of the BluBird system. Work is presently under way to test adjustments to the Gaussian plume model for different conditions, and to implement a Gaussian puff approach (e.g., [55]), which can improve localization and provide more options for enhanced physical treatments.

Finally, we note the difference between how probability of detection is defined for CEMS based on programs such as ADED versus how the same statistic is applied to periodic survey technologies. We also suggest that the definition of probability of detection, and how it is applied, should be expanded to consider factors in addition to emission rate. These could include PoD, as a function of emission duration, or perhaps even PoD within a given cost constraint that defines the number of sensors, and/or type of sensors, that can be deployed on a site.

## Figures and Tables

**Table 1:** Coefficients used to calculate $\sigma_{y}$ and $\sigma_{z}$.

Pasquill Stability Class	*A_y_*	*B_y_*	*A_z_*	*B_z_*
A	0.243	2.0793	0.1444	−0.1015
B	0.2102	1.6724	0.1218	0.2247
C	0.1767	1.2893	0.0993	0.5491
D	0.1462	0.9953	0.0838	0.5174
E	0.1156	0.7217	0.0684	0.4828
F	0.096	0.5934	0.0546	0.4741
G	0.0763	0.4556	0.0409	0.4608

**Table 2:** Subsets of METEC ADED 2024 results used for analysis, along with “potential” classification options for TP, FN and FP determination. “V.3” refers to the ADED data file version provided by METEC.

Classification Criterion	Explanation
METEC standard	Full METEC v.3 results data set with METEC-assigned classifications
30-min	Full METEC v.3 results data set with a 30-min early-start time threshold used for classification
75% overlap	Full METEC v.3 results data set with a requirement that individual METEC releases and BluBird detections overlap in time by ≥75%

**Table 3:** Detection classification results for ADED 2024 experiments, including potential detections (per-experiment set; 347 cases). “Rescues” are reports that were classified as FPs but that transitioned to TPs using the different “potential” classification criteria.

Classification Criterion	# of TPs	# of FNs	TP%	Rescues *
METEC standard (20-min start threshold)	314	33	90.5	0
30-min start threshold	325	22	93.7	13
75% overlap	338	9	97.4	36

***Notes.* #**—number of occurrences in the indicated category. *—“Rescues” is the count of METEC-standard FN classifications that were reclassified as TP under the alternative classification criterion; by construction, the METEC standard criterion has zero rescues.

**Table 4:** Detection classification results for ADED 2024 all releases, including potential detections (all-release set; 775 cases).

Classification Criterion	# of TPs	# of FNs	TP%
METEC standard (20-min start threshold)	536	239	69.2
30-min start threshold	549	226	70.8
75% overlap	572	203	73.8

***Notes.* #**—number of occurrences in the indicated category.

**Table 5:** Detection classification results for ADED 2024 single-release experiments, including potential detections (single-release set; 103 cases). “Rescues” are reports that were classified as FPs but that transitioned to TPs using the different “potential” classification criteria.

Classification Criterion	# of TPs	# of FNs	TP%	Rescues *
METEC standard (20-min start threshold)	88	15	85.4	0
30-min start threshold	95	8	92.2	7
75% overlap	99	4	96.1	11

***Notes.* #**—number of occurrences in the indicated category. *—“Rescues” is the count of METEC-standard FN classifications that were reclassified as TP under the alternative classification criterion; by construction, the METEC standard criterion has zero rescues.

**Table 6:** Detection classification results for ADED 2022 per-experiment and all-releases sets. For the 2022 data, use of the different classification criteria listed had no effect.

Subset	Classification Criterion	# of Cases	# of TPs	# of FNs	TP%
Per-experiment	METEC standard (20-min start threshold)	325	184	141	56.6
All-releases	METEC standard (20-min start threshold)	557	194	363	34.8

***Notes.* #**—number of occurrences in the indicated category.

**Table 7:** Trivariate-at-median 90% PoD (kg/h) by campaign, results subset, and detection criterion, as a function of release rate. (* Rate is outside the program release-rate range). PoDs for the potential detections using the alternative classifications are included for sensitivity-comparison purposes.

Campaign	Subset	90% PoD (METEC Standard)	90% PoD (30-min Start-Gap)	90% PoD (75% Overlap)
ADED 2024 (BluBird 2.0)	Per-experiment	0.64 kg/h	0.38 kg/h	0.25 kg/h
	Single-release	1.59	0.49	0.26
	All-releases	16.45 *	9.04	3.94
ADED 2022 (BluBird 1.5)	Per-experiment	26.88 *	26.88 *	26.88 *
	Single-release	13.43 *	13.43 *	13.43 *
	All-releases	8.95	8.95	8.95

**Table 8:** Statistics associated with PoD curves in Figure 14, with the alternative classification potential detections included for comparison, for the all-release subset (top) and per-experiment subset (bottom).

All-release subset (*n* = 775 individual releases)						
**Criterion**	***n***	***n* TP**	**Point POD90 (kg/h)**	**Delta-Method 95% CI**	**Bootstrap Median**	**Bootstrap 95% CI**
METEC standard (20 min)	775	536	16.44	2.65–101.9	16.34	4.62–436.4
30-min start-gap	775	549	9.04	2.32–35.1	9.20	3.31–73.5
75% overlap	775	572	3.94	1.62–9.57	3.87	1.89–17.6
Per-experiment (at-least-one-release) subset (*n* = 347 experiments; rate = SUM)						
Criterion	*n*	*n* TP	Point POD90 (kg/h)	Delta-Method 95% CI	Bootstrap Median	Bootstrap 95% CI
METEC standard (20 min)	347	314	0.640	0.176–2.33	0.578	0.00–1.58
30-min start-gap	347	325	0.380	0.089–1.63	0.357	0.00–0.80
75% overlap	347	338	0.251	0.132–0.481	0.240	0.061–0.371

**Table 9:** Summary of emission rate errors for per-experiment paired TP releases [(BluBird − METEC)/METEC].

Emission Rate Range (kg/h)	# Cases (*n*)	Median Relative Error	Interquartile Range (IQR; p25 to p75)
0.0 to 0.5	27	−31%	[−71%, +67%]
0.5 to 2.0	189	−58%	[−75%, −1%]
2.0 to 3.0	28	−39%	[−71%, −24%]
3.0 to 7.0	64	−66%	[−81%, −45%]
7.0 to 10.0	6	−79%	[−86%, −69%]

**Table 10:** Percent of BluBird-estimated emission rates within specified ranges of METEC rates for the per-experiment and single-release data sets (METEC standard grading).

Within Factor of:	Per-Experiment (*n* = 314)	Single-Release (*n* = 88)
1.25 (±25%)	9%	17%
1.5	20%	23%
2	37%	43%
3	56%	67%

## Data Availability

The original contributions presented in this study are included in the article/Supplementary Material. Further inquiries can be directed to the corresponding author.

## Supplementary Materials

The following supporting information can be downloaded at: https://www.mdpi.com/article/10.3390/s26185946/s1, The original METEC data files resulting from Earthview’s participation in ADED 2022 and ADED 2024, with derived columns for overlap, Jaccard, and alternative-criterion classifications, are provided in File S1 (containing ADED_2022_supplementary_data.xlsx and ADED_2024_v3_supplementary_data.xlsx). Also included are detection-and-report overlay plots spanning the full ADED 2024 period.
